# Metabolomic Fingerprints of Individual Algal Cells Using the Single-Probe Mass Spectrometry Technique

**DOI:** 10.3389/fpls.2018.00571

**Published:** 2018-04-30

**Authors:** Mei Sun, Zhibo Yang, Boris Wawrik

**Affiliations:** ^1^Department of Chemistry and Biochemistry, University of Oklahoma, Norman, OK, United States; ^2^Department of Botany and Microbiology, University of Oklahoma, Norman, OK, United States

**Keywords:** *Scrippsiella trochoidea*, phytoplankton, nutrient limitation, nitrogen, marine algae, metabolomics, single-cell analysis

## Abstract

Traditional approaches for the assessment of physiological responses of microbes in the environment rely on bulk filtration techniques that obscure differences among populations as well as among individual cells. Here, were report on the development on a novel micro-scale sampling device, referred to as the “Single-probe,” which allows direct extraction of metabolites from living, individual phytoplankton cells for mass spectrometry (MS) analysis. The Single-probe is composed of dual-bore quartz tubing which is pulled using a laser pipette puller and fused to a silica capillary and a nano-ESI. For this study, we applied Single-probe MS technology to the marine dinoflagellate *Scrippsiella trochoidea*, assaying cells grown under different illumination levels and under nitrogen (N) limiting conditions as a proof of concept for the technology. In both experiments, significant differences in the cellular metabolome of individual cells could readily be identified, though the vast majority of detected metabolites could not be assigned to KEGG pathways. Using the same approach, significant changes in cellular lipid complements were observed, with individual lipids being both up- and down-regulated under light vs. dark conditions. Conversely, lipid content increased across the board under N limitation, consistent with an adjustment of Redfield stoichiometry to reflect higher C:N and C:P ratios. Overall, these data suggest that the Single-probe MS technique has the potential to allow for near *in situ* metabolomic analysis of individual phytoplankton cells, opening the door to targeted analyses that minimize cell manipulation and sampling artifacts, while preserving metabolic variability at the cellular level.

## Introduction

Globally, marine phytoplankton contribute ca. 45 petagrams carbon per annum to net primary production (NPP) (Falkowski et al., 1998). Phytoplankton are thus important drivers of several global biogeochemical cycles, notably of elements that are components of cellular biomass, including carbon (C), nitrogen (N), phosphorus (P), silicate (Si), and others. The abundance of phytoplankton in marine systems is shaped by the availability of nutrients as well as physical oceanic processes. Typically, phytoplankton biomass can be broadly approximated from environmental variables, such as nutrient concentrations, sea surface temperature, and solar irradiance (Antoine et al., 1996; Falkowski et al., 1998). However, at a more granular level, such as the cellular response of individual phytoplankton to dynamic oceanographic conditions, requisite adaptations are often not well-understood. In particular, whether phytoplankton are nutrient limited in the environment has long attracted the attention of oceanographers who are trying to understand the controls on NPP, given the large impact that limitation may have on the structure of marine ecosystems.

Historically, both N and P have been understood to, at times, limit productivity in marine systems (Howarth, 1988), but other nutrient, such as iron (Coale et al., 1998; Landry et al., 2000), also appear to play an important role. Additionally, phytoplankton can be co-limited by more than one nutrient due to their limited absolute abundance (i.e., kinetic limitations), or the acquisition of one nutrient may be dependent on the concentration of another (Saito et al., 2008). Understanding requisite limitations in natural systems is unfortunately not a straightforward matter, given the restricted methodological options available to researchers. For example, nutrient limitation is frequently invoked via nutrient ratios (Liebig’s law of the minimum), yet this ignores that turnover rates can be high in the face of low, but non-limiting ambient concentrations (Beardall et al., 2001; Wawrik et al., 2004). Similarly, bottle incubations (spiking nutrients) are used to infer nutrient limitation via measurements of cellular activity (e.g., carbon fixation), biomass (e.g., chl *a*), or photosynthetic capacity (F_v_/F_m_), but interpretation of requisite data can be challenging, given that measurements are made on bulk communities in which individual phytoplankton species may exhibit differential behavior. Changes in elemental uptake ratios (C: P or C: N) have been used to infer nutrient limitation (Rees et al., 1995; Beardall et al., 2001), but such analyses suffer from drawbacks similar to those of bottle incubations, in that measurements are conducted on bulk communities.

An alternate avenue has been the development of specific molecular probes for the expression of marker genes. This approach draws on a long tradition of studies that aim to develop molecular targets for specific microbial nutrient cycling activities in the environment (Scala and Kerkhof, 1998; Allen et al., 2001; Wawrik et al., 2002; and many more). For example, the expression of the global nitrogen regulator NtcA in marine cyanobacteria has been used to assess the nutritional status of natural populations of *Synechococcus* (Lindell and Post, 2001). Similarly, the expression of the *nifH* gene is widely distributed in marine systems (Turk et al., 2011), indicator of cellular alleviation of N limitation in some microbial populations. However, while this approach is powerful, it typically requires a fairly good understanding of the underlying molecular mechanisms and genetic diversity of related genes to allow for the derivation of probes or primers. Other studies have applied less targeted, transcriptomic approaches to detect nutrient limitation in marine phytoplankton (Hwang et al., 2008; Cooper et al., 2016; Harke et al., 2017), but analogous studies are more difficult in diverse natural assemblages for which genetic information is not necessarily available. Given this, our understanding of the degree and severity of nutrient limitation in the marine environment, especially at the induvial species and cellular level, remains poorly constrained.

Recently, several approaches have been developed to perform the single cell analysis. These include fluorescence, capillary electrophoresis, and mass spectrometry (MS) (Woods and Ewing, 2003; Woods et al., 2004; Cohen et al., 2008; Urban et al., 2011; Cahill et al., 2015). Here, we report the application of a novel technology that allows for the analysis of the metabolome of single phytoplankton cells to assess their physiological status. The approach utilizes the ‘Single-probe,’ a micro-scale sampling and ionization device, that is coupled to an XYZ-stage to directly insert into single phytoplankton cells to extract cellular metabolites for real-time MS analysis (Pan et al., 2014; Rao et al., 2016b). Our aims were threefold. First, we intended to establish a proof of concept, i.e., demonstrate that metabolome data could be generated for single marine phytoplankton cells. Second, we aimed to demonstrate that physiological changes at the cellular level could be detected via analysis cellular metabolites sampled. Lastly, we aimed to demonstrate that single cell metabolomics can be utilized to assess whether cells are experiencing different illumination levels and nutrient limitation.

## Materials and Methods

### Cultures

Non-axenic *Scrippsiella trochoidea* CCMP 3099 was originally obtained from the National Center for Marine Algae and Microbiota (Provasoil-Guillard NCMA, Boothbay Harbor, ME, United States). For maintenance, cultures were grown in L1 seawater media (Guillard and Ryther, 1962; Guillard et al., 1973; Guillard and Hargraves, 1993). Medium was prepared from natural seawater collected near Key West (salinity of 33), which was aged for at least 6 month in the dark and autoclaved. Maintenance and experimental cultures were grown in a light/dark incubator at 23–24°C and 30–40 μmol quanta⋅m^-2^ s^-1^ light under a 12-h light:12-h dark cycle.

### Experimental Culture Setup

For the light/dark comparisons, cultures were grown under replete conditions in full L1 media containing 880 μM NaNO_3_ and 36 μM NaH_2_PO_4_ (N/P ratio 24:1). Experimental cultures were started as a 1:10 inoculum from exponentially growing cultures into 1 L of media in 2.5 L Pyrex Fernbach flasks without shaking and monitored daily via cell counts and chlorophyll measurements. Cell counts were conducted by addition of 1% Lugol’s iodine and direct counting of cells in 96-well microtiter plates using a dissection microscope. Dilutions were made as necessary and at least 10 wells containing 100 μL diluted culture were counted to average cell counts. Chlorophyll *a* was measured via fluorometry (Welschmeyer, 1994) by filtering 5 mL of culture in triplicate onto GF/F filters, over-night extraction with methanol, and quantification using a Turner Trilogy Laboratory Fluorometer. Cultures were grown into late-log phase (data not shown) and then sampled 3 h before and 3 h after the light was turned on. Cultures were sub-sampled for MS analysis, making sure to keep ‘dark’ sample exposure to light to a minimum by wrapping sampling tubes in aluminum foil.

N-deplete cultures were generated by first growing cells on L1 media with an N:P ratio of 2.4:1 (88 mM nitrate: 36 mM phosphate) analogous what has been previously described (Harke et al., 2017). This lower ratio stoichiometrically limited cultures in nitrogen and at least three transfers were performed to ensure no carryover from higher nutrient full L1 medium. Cultures were monitored daily via cell counts (see above), chlorophyll *a* quantification (see above), and quantification of nitrate/nitrite via a Vanadium reduction method (Miranda et al., 2001). Parallel cultures were set up in which one culture was allowed to run out of nitrogenous nutrients (N-deplete), while the culture (control) was fed additional nitrate every second day to bring total nitrate/nitrite concentrations back to starting levels (Supplementary Figure S1). Once nitrate/nitrite levels dropped below the limit of detection (∼1–2 μM) in the N-deplete culture, cultures were grown for an additional 24 h before sampling to ensure that N-depletion was complete. Both the N-deplete and replete cultures were then sampled for MS analysis of single cells.

### Mass Spectrometry

**FIGURE 1:**
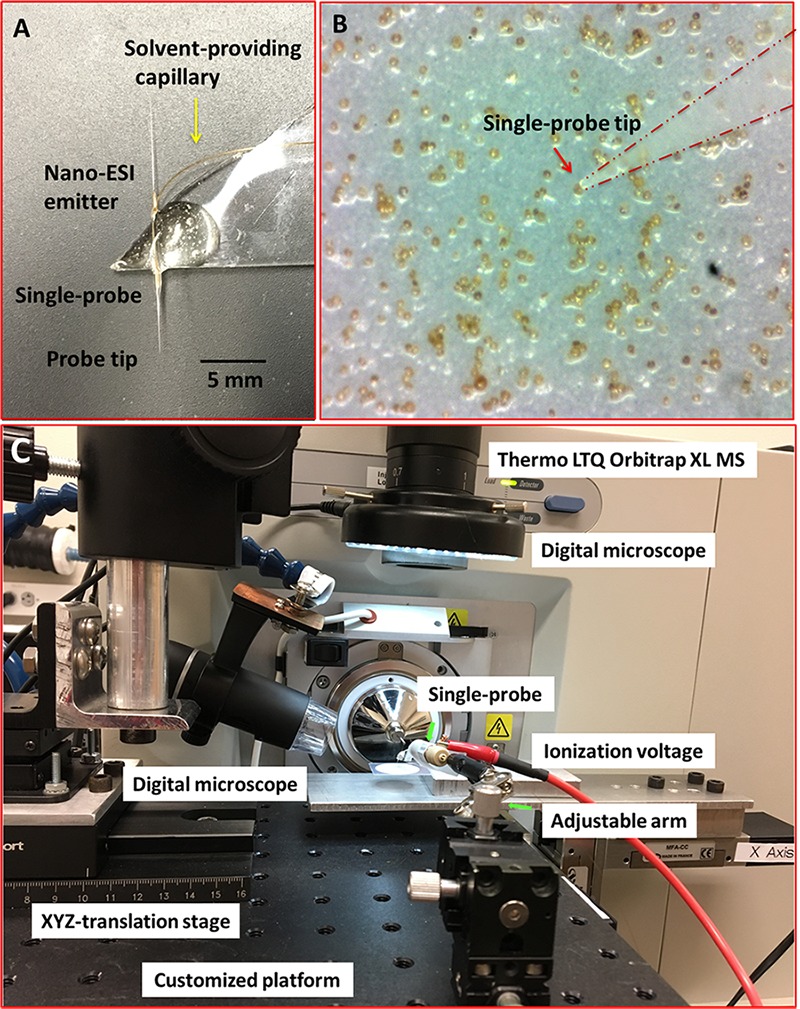
Experimental setup to measure single *Scrippsiella trochoidea* cells using the “Single-probe” MS techniques. **(A)** Photograph of the Single-probe device with its different components labeled; **(B)** image from microscope-linked camera used to target single *S. trochoidea* cell with the Single-probe; **(C)** setup used to manipulate the Single-probe MS device with components labeled.

Individual cells of *S. trochoidea* were analyzed via the ‘Single-probe’ MS techniques. Detailed fabrication protocols of the Single-probe have been previously described (Pan et al., 2014; Rao et al., 2015; Sun et al., 2017). Briefly, a Single-probe (**Figure 1A**) has three components: a dual-bore quartz tubing [outer diameter (OD) 500 μm; inner diameter (ID) 127 μm, Friedrich & Dimmock, Inc., Millville, NJ, United States] pulled using a laser pipette puller (P-2000 micropipette puller, Sutter Instrument, Novato, CA, United States), a fused silica capillary (OD 105 μm; ID 40 μm, Polymicro Technologies, Phoenix, AZ, United States), and a nano-ESI emitter made from the same type of fused silica capillary. A Single-probe is fabricated by embedding a fused silica capillary and a nano-ESI emitter into both of the channels of the laser-pulled dual-bore quartz needle.

For the analysis, cells were deposited onto 0.2 μm polycarbonate membranes by gentle filtration, and the cells were rinsed with phosphate buffered saline (PBS; 137 mM NaCl, 2.7 mM KCl, 4.3 mM Na_2_HPO_4_, 1.47 mM KH_2_PO_4_; pH of 7.4) to remove culture medium. Filters were then placed on a home-built XYZ-translation stage system and spatial motion was controlled by a custom designed LabView software package (Lanekoff et al., 2012). The Single-probe tip (<10 μm) was then precisely insert into single *S. trochoidea* cells (typically ∼20–30 μm cellular diameter) using a microscope as a guide (**Figure 1B**). During the experiment, a syringe (250 μl; Hamilton, Co., Reno, NV, United States) was used to continuously provide the sampling solvent (acetonitrile; Sigma-Aldrich, St. Louis, MO, United States), and a liquid junction formed at the Single-probe tip to perform highly efficient extraction of cellular contents. The analytes were withdrawn by capillary action toward the nano-ESI emitter, and ionized for analysis using a Thermo LTQ Orbitrap XL mass spectrometer (Thermo Scientific, Waltham, MA, United States) (**Figure 1C**). Mass analyze parameters were as follows: mass resolution 60,000, +4 kV ionization voltage at positive ion mode (0.05–0.07 μA of ion current), 1 microscan, 100 ms max injection time, and automatic gain control on.

### Data Analysis

The Thermo Xcalibur Qual Browser (Thermo Scientific, Waltham, MA, United States) was used to export MS data (m/z values with relative intensities) as tab-delimited data files. As a conservative approach, only relatively abundant peaks with ion intensities > 10^3^ were exported. This approach excluded 6% of low signal peaks as background while retaining 94% of total signal intensity. The relative ion intensities were normalized to the total ion current to minimize the influence induced by fluctuations of ion signals during experiments. The Geena 2 online software tool^1^ was then used for peak alignment (Romano et al., 2016), and the aligned m/z values were then used for comparisons. Parameters used in Geena 2 include analysis range (from 100 to 1500 m/z), maximum number of isotopic replicas (5), maximum delta between isotopic peaks (0.05 Da), and maximum delta for aligning replicates (0.01 Da). Metaboanalyst 3.0^2^ was used to conduct statistical data analysis, including PLS-DA (partial least squares discriminant analysis) and *t*-tests (Xia et al., 2015; Xia and Wishart, 2016). PLS-DA was used to visualize differences in chemical composition profiles among treatment groups, while *t*-tests were applied to extract molecular peaks with significant abundance changes (*p* < 0.05). Finally, the online database METLIN^3^ was used to tentatively label all ions of interest (Smith et al., 2005; Guijas et al., 2018), to perform hierarchical clustering, and to generate heat maps. Lastly, Pathos^4^ was used to attempt identification of significantly regulated metabolic pathways by considering all KEGG maps in all organisms (Leader et al., 2011).

## Results

The main aims of this study were to develop a single-cell-based metabolomic methodology that could be applied to individual algal cells and to demonstrate that this technology can detect physiological responses to environmental stimuli. The described setup allowed us to sample individual cells by targeting them with the tip of the Single-probe (**Figure 1B**). Our ‘proof of concept’ experiment involved a comparison of cells in the context of diurnal illumination changes, which are known to induce significant changes in algal cellular metabolomes (Vidoudez and Pohnert, 2012).

**FIGURE 2:**
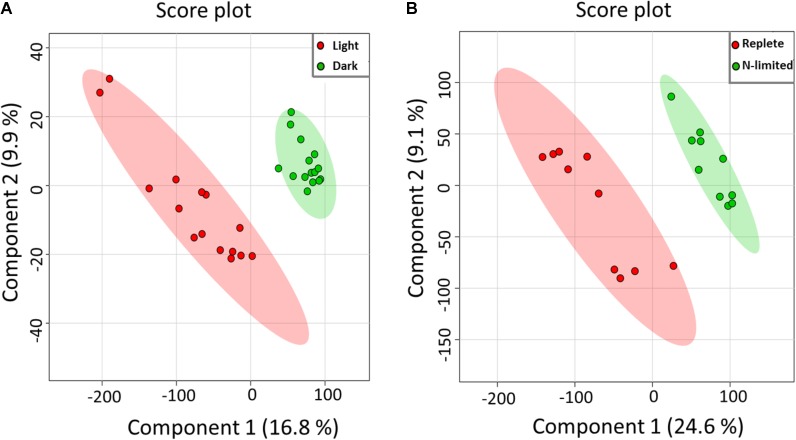
Partial least aquares discriminant analysis (PLS-DA) of MS data. All detected metabolites were analyzed, visualizing the overall effect on the metabolome of single *S. trochoidea* cells by **(A)** light vs. dark conditions [results are reported from 15 replicates (*n* = 15) in each group], and **(B)** N-limited vs. replete conditions (*n* = 10 for each group).

Using the Single-probe MS technique, distinct and clear differences were observed in the *S. trochoidea* metabolome under different light levels. A total of 1,085 and 1,103 peaks were detected under light and dark conditions, respectively. We have tentatively labeled 581 species (i.e., 558 metabolites and 23 peptides; Supplementary Table S1). All MS data are archived using Zenodo (DOI: 10.5281/zenodo.1188486), and the descriptions of MS files were summarized in Supplementary Table S2, and a single cell mass spectrum (Supplementary Figure S2) is shown as an example. Of these detected species, 306 and 321 were differentially abundant among treatments (*t*-test; *p* < 0.05) under light and dark conditions, respectively. Results obtained from PLS-DA (**Figure 2A**) revealed that metabolic features formed distinct clusters (15 cells in each group), and this difference was highly significant (*p* = 5 × 10^-4^; permutation test in Metaboanalyst 3.0). Cross-validation of the PLS-DA model was conducted, and our results (Q^2^ > 0.5) indicate this model provided a good predictability without overfitting (Supplementary Table S3) (Worley and Powers, 2013; Triba et al., 2015). To investigate which metabolic pathways may have been significantly impacted by the difference in illumination levels, all ions with significant differences (*p* < 0.05) were selected and tentatively labeled by searching their m/z values in Pathos, considering all KEGG metabolites, and retaining pathways for which at least two hits were observed (**Table 1**). A criterion of two hits to an individual pathway was used, because the reliability of assignments for metabolites based on m/z values alone is limited. Most strikingly, only 12 (5%) and 16 (19%) of differentially abundant metabolites could be assigned to KEGG pathways for light and dark conditions respectively, indicating that the majority of the metabolic response to light changes in *S. trochoidea* is not captured in KEGG metabolic maps. With respect to pathways with at least two hits (**Table 1**), light favors biosynthesis of molecules potentially linked to the production of 12-, 14-, and 16-membered macrolides. In particular, several hits in the pathway for Avermectin were observed (**Table 1A**). Under dark conditions, identified pathways included those for polyketides, porphyrin, chlorophylls, terpenoids, and limonene (**Table 1B**). With respect to the four hits in porphyrin and chlorophyll metabolism, all were linked to the production of phycobillins.

**Table 1:** Pathways containing more than one metabolite with significantly different abundance under **(A)** illuminated conditions compared to cultures during **(B)** dark condition.

Pathway	# of Metabolites	Metabolite	Counter-ion
**(A)**			
Biosynthesis of 12-, 14-, and 16-membered macrolides	7	6-Deoxyerythronolide B (C_21_H_38_O_6_)^∗^	(+Na^+^)
		Avermectin A2a (C_49_H_76_O_15_)^∗^	(+H^+^)
		Avermectin A2a aglycone (C_35_H_52_O_9_) ^∗^	(+Na^+^)
		Avermectin B2b (C_47_H_72_O_15_) ^∗^	(+Na^+^)
		Demethyllactenocin (C_37_H_61_NO_14_) ^∗^	(+NH_4_^+^)
		Erythronolide B (C_21_H_38_O_7_) ^∗^	(+K^+^)
		L-Oleandrosyl-oleandolide (C_27_H_46_O_10_) ^∗^	(+Na^+^)

**(B)**			
Porphyrin and chlorophyll metabolism	4	(3Z)-Phytochromobilin (C_33_H_36_N_4_O_6_)	(+Na^+^)
		15,16-Dihydrobiliverdin (C_33_H_36_N_4_O_6_)	(+Na^+^)
		Bilirubin (C_33_H_36_N_4_O_6_)	(+Na^+^)
		I-Urobilinogen (C_33_H_44_N_4_O_6_) ^∗^	(+K^+^)
Biosynthesis of type II polyketide products	2	19-Hydroxytetrangulol (C_19_H_12_O_5_)	(+K^+^)
		Dehydrorabelomycin (C_19_H_12_O_5_)	(+K^+^)
Limonene and pinene degradation	2	(3R)-3-Isopropenyl-6-oxoheptanoate (C_10_H_16_O_3_)	(+NH_4_^+^)
		(3S)-3-Isopropenyl-6-oxoheptanoate (C_10_H_16_O_3_)	(+NH_4_^+^)
Monoterpenoid biosynthesis	2	1,6,6-Trimethyl-2,7-dioxabicyclo[3.2.2]nonan-3-one (C_10_H_16_O_3_)	(+NH_4_^+^)
		4,5-Dihydro-5,5-dimethyl-4-(3-oxobutyl)furan-2(3H)-one (C_10_H_16_O_3_)	(+NH_4_^+^)

*Metabolites were analyzed using Pathos (http://motif.gla.ac.uk/Pathos/pathos.html) by considering all KEGG maps in all organisms under positive ion mode. ^∗^Denotes that the formula is unique among all metabolites in KEGG maps.*

Given the successful proof of concept for light/dark conditions, we aimed to investigate whether single cell metabolomic analysis might be utilized to investigate the nutritional status of individual phytoplankton cells. As shown in **Figure 2B**, PLS-DA results indicated that single cellular metabolomes of *S. trochoidea* under N-limited condition were clearly different from those in nitrogen replete cells (*n* = 10). As with the light–dark treatments, N limitation induced a highly significant response (*p* = 1 × 10^-3^ and Q^2^> 0.5; Supplementary Table S3). KEGG pathway analysis was attempted, but yielded no metabolites in the significantly upregulated pool that could be matched to KEGG. Similarly, only 8 (<4.5%) metabolites from the down-regulated pool could be mapped with KEGG and no pathways contained more than a single hit. Little information could therefore be gleaned about requisite physiological responses via KEGG analysis, and an alternative approach to analysis was therefore taken. First, we hypothesized that N-limitation should be reflected in the C/N and N/P rations of the cellular metabolome, as cells might physiologically adjust to environmental conductions by choosing cellular metabolites with lower N content (Beardall et al., 2001; Geider and La Roche, 2002). Second, metabolites were analyzed for the putatively detected N-containing lipids (e.g., phosphoethanolamine), given that physiological responses are often manifest in the lipid pool.

**FIGURE 3:**
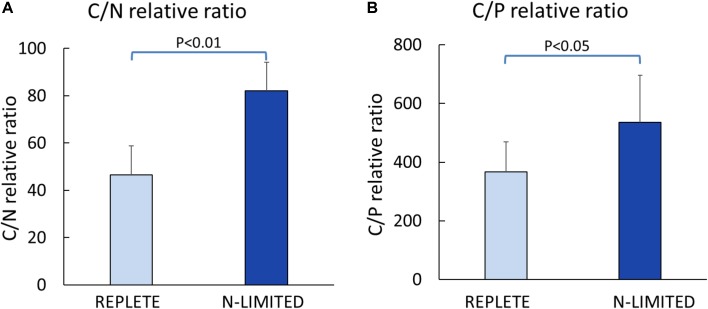
Elemental ratios of significantly regulated metabolites. **(A)** Carbon to Nitrogen ratio; **(B)** Carbon to Phosphorus ratio.

**FIGURE 4:**
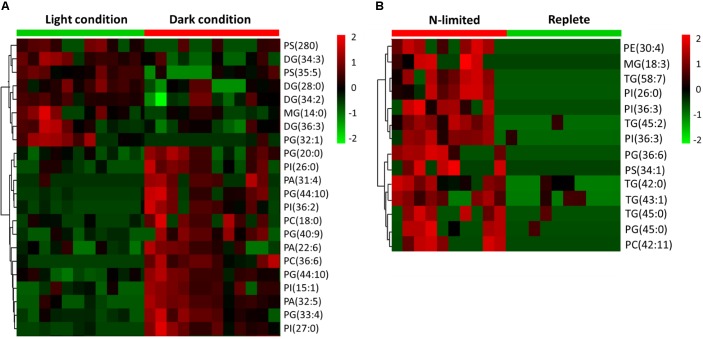
Heat maps generated from hierarchically clustering summarizing the cellular lipids measured from single *S. trochoidea* cell under different light and nutrient conditions. Only lipids for which abundances were significantly different among treatment pairs are shown. Shown are differences for **(A)** light vs. dark conditions and **(B)** replete vs. nitrogen (N) limiting conditions. Red indicates elevated and green indicates decreased signal with respect to the mean signal observed for all tested cells. PA, phosphatic acid; PE, phosphoethanolamine; PG, phosphatidylglycerol; PS, phosphatidylserine; PI, phosphatidylinositol; PC, phosphatidylcholine; MGs, monoglycerides; DGs, diglycerides; TG, triglyceride.

C: N and C: P ratios were calculated by summing the product of the number of C, N, P atoms in each metabolite with its relative abundance across all metabolites (**Figure 3**). This analysis reveals that N-limited *S. trochoidea* has significantly higher C:N ratios in regulated metabolites as compared to replete conditions (*p* = 4 × 10^-6^) (**Figure 3A**). Similarly, C:P ratios were affected and were significantly higher under N-limitation (*p* < 0.044) (**Figure 3B**). Concurrently, the abundances of at least some cellular lipids were significantly affected. When all significantly regulated metabolites putatively identified as lipids are considered (**Figure 4**), it appears that the availability of light correlates with both up- and down-regulation of specific lipid complements (**Figure 4A**). Under N-limitation, however, a significant decrease of lipid abundances is observed (*p* < 0.05) (**Figure 4B**).

## Discussion

Historically, the majority of oceanographic research has targeted natural populations of phytoplankton via bulk filtration techniques (e.g., filtration onto GF/F filters) to assess physiological responses to environmental factors such as light or nutrient limitation. While much has been learned using bulk filtration, requisite approaches suffer from important limitations. Most notably, individual populations or different species are not adequately resolved in this manner. It is also now well-appreciated that cellular functions, such as gene expression, proliferation, or programmed cell death, are subject to significant stochasticity, leading to high cellular chemical and phenotypic diversity at the single cell level, potentially obscuring some important patterns (Fagerer et al., 2013; Comi et al., 2017; Guillaume-Gentil et al., 2017). Single-cell analysis is therefore an attractive methodological choice when studying rare types of cells (cells available are inadequate for bulk analysis) or cells in heterogenous populations, where cell separation or sorting are impractical. Here we present the first report of a single cell-based metabolomic technology that allows for the analysis of intracellular intermediates of individual phytoplankton cells. The Single-probe directly collects cellular contents of living cells, and it does this without significant sample preparation steps (e.g., filtration or solvent extraction), thereby allowing for real-time and targeted analysis that minimizes sampling artifacts.

We note that bulk analyses [e.g., liquid chromatography (LC)/MS, gas chromatography (GC)/MS), or direct-injection MS without separation] were not conducted in this study. It is likely that the number and the types of metabolites detected here differs from those that might have been observed in bulk measurements. Traditional LC/MS and GC/MS techniques have certainly been used in the analysis of marine algae (Barofsky et al., 2009; Burriesci et al., 2012), and their value is clear in requisite studies. However, a direct comparison was not conducted, because we do not view the use of the Single-probe as a replacement for more traditional bulk biomass approaches. Rather, single-cell analysis can serve as a complementary method that allows exploration of inherent cell-to-cell variability in complex and heterogeneous systems that may not be resolved using more traditional approaches.

Given the tip size of the Single-probe, which is ca. 10 μm in diameter, analysis is currently limited to larger phytoplankton and protists. However, the currently applicable size range includes many important bloom forming algae, including toxin producing genera such as *Karenia* (20–40 μm) or *Pseudo-nitzschia* (40–175 μm). The Single-probe might therefore offer unique opportunities to help understand the biological forces that shape the success of these organisms in an ecosystem by revealing their metabolomic responses to changing environmental conditions or experimental treatments at the cellular level. We note that the expensive and bulky configuration of equipment described here still precludes easy field deployment, making the Single-probe MS technique most useful under conditions where direct access to the lab is available, or when experimental cultures are assayed. Further development in the miniaturization of high-resolution mass spectrometer or sample preservation maybe be helpful in this regard. In addition, due to the limited amount of cytoplasm found in an individual cell and limitation on the achievable sensitivity of mass spectrometers, most single cell MS studies to date are primarily focused on the analysis of relatively small molecules such as metabolites and peptides (Rubakhin et al., 2011). The detection of larger molecules, such as proteins presenting as relatively lower abundances, at single cell level remains very challenging.

Despite these limitations, single-cell MS technique holds great promise for environmental research. The Single-probe MS technique has been successfully used to study live single cancer cells (Pan et al., 2014, 2016), to map biomolecules on animal tissues with high spatial resolutions (Rao et al., 2015, 2016a,b), and to analyze the extracellular metabolites inside spheroids (Sun et al., 2017). With respect to the ability to detect a large range of cellular metabolites from single cells (i.e., from only a few pico-litters of cytoplasm sampled from a cell with a diameter of approximately10 μm), the Single-probe MS setup provides excellent detection sensitivity. We note, however, that single cell MS measurements are not strictly repeatable (cellular contents are consumed in each measurement), and that it is impractical to evaluate reproducibility in this regard. Multiple cells must therefore be measured (e.g., *n* = 10–15 in each group in the current study) to normalized intensities for statistical data analysis and to minimize the influence of uncertainties such as fluctuation of ion signals, minor changes of experimental tuning conditions, instrument noise, and variances associated with the matrix effect.

Here, were extend these advances by demonstrating that the physiological status of phytoplankton with respect to light and nutrients can be assessed. The availability of light was correlated with a highly significant response in the metabolite profile of *S. trochoidea* cells (**Figure 2**). As might be expected, metabolites related to porphyrin and chlorophyll pathways stood out. In particular, the detected metabolites are related to phytochrome metabolism, which is consistent with the notion that phytochromes are signal-transducing photoreceptors (Smith, 2000). Beyond phototaxis and a handful of secondary metabolite intermediates, however, few metabolites could be assigned to KEGG pathways. This, perhaps, speaks to our limited understanding of metabolism in Dinoflagellate algae. Transcriptomic analysis of *S. trochoidea* (same strain used here) indicated that this species makes perhaps in excess of 10^5^ transcripts, the majority of which could not be annotated (Cooper et al., 2016).

Cellular lipids have been used to parse physiological responses of living cells under different illumination conditions (Chen et al., 2015; Wacker et al., 2016). For example, under dark conditions, mRNA for some genes involved in lipid biosynthesis have been observed to be elevated in *Chlorella* (Chen et al., 2015). Differential responses of lipid abundance have also been observed in the marine dinoflagellate *Prorocentrum minimum* under dark vs. light conditions (Manoharan et al., 1999), and light-induced significant changes in the fatty acid profiles have been reported for freshwater diatom, chrysophyte, cryptophyte, and zygnematophyte algae (Wacker et al., 2016). Differences in requisite light-dependent cellular lipid profiles are thought to be related to alterations of energy storage and the compositions of chloroplast membranes (Guihéneuf et al., 2009; Trentacoste et al., 2013; d’Ippolito et al., 2015). Consistently, our experiments indicated both up- and down-regulation of lipids in response to light in *S. trochoidea*. The pattern of changes in lipid composition in response to N-limitation was, however, quite different from that observed under light limitation. Most notably, all differentially regulated lipids were more abundant under the nutrient limiting conditions. This is potentially the result of limitation induced imbalances in cellular elemental composition. Under N-limitation almost all parts of central metabolism are impacted (and potentially slowed due to limiting resources), yet lipids, for the most part, do not contain N atoms and their biosynthesis might thereby proceed so long photosynthesis can proceed. Whether lipid accumulation in *Scrippsiella* is adaptive, however, by possibly allowing energy storage to gain advantages when nutrients are more readily available, or is simply a consequence of the onset of senescence remains to be investigated. Changes in the C:N and C:P ratios do appear to be reflected in the metabolome overall (**Figure 3**). This observation is consistent with the notion that elemental ratios have considerable plasticity based on nutrient availability (Geider and La Roche, 2002). Previous studies have suggested that nutrient deficiency can cause the accumulation of lipids, such as triglyceride (Takagi et al., 2000; Xin et al., 2010; El-Kassas, 2013), and that phytoplankton funnel excess NADPH to the biosynthesis of triglyceride and fatty acids to regenerate NADP^+^ (Thompson, 1996; Hu et al., 2008; El-Kassas, 2013). Correspondingly, we observed that a number of triglycerides significantly increased in the N-limited group (**Figure 3B**). We also note that C:N and C:P ratios reported here deviate from Redfield’s expectations (C:N:P ∼ 106:16:9) (**Figure 3A**). However, these observations are consistent with our approach. The elemental Redfield ratios are based on all cellular components, yet data analyzed here only covers a subset of all cellular constituents. For example, the Single-probe MS techniques cannot currently measure large biomolecules such as proteins and nucleic acids which are N-rich. The absence of nucleic acids in calculations likely leads to high C:P ratio estimates (Maske, 1982; Laws et al., 1983; Geider and La Roche, 2002). Values reported here are therefore not inconsistent with generally expected elemental ratios for phytoplankton biomass.

In summary, we report the development of a methodology for single-cell metabolomics of small protists such as marine dinoflagellate algae. Using the Single-probe MS technology we can efficiently monitor the cellular physiological responses of phytoplankton under different illumination and nutrient conditions. This offers the opportunity for real-time analysis of natural populations and has the potential ability to provide information about the dynamic metabolic response of individual cells to environment stimulation.

## Author Contributions

BW provided funding, conducted culture work, and contributed to data analysis and writing. ZY provided funding, was involved in MS work, and contributed to data analysis and writing. MS conducted much of the work and contributed to data analysis and writing.

## Conflict of Interest Statement

The authors declare that the research was conducted in the absence of any commercial or financial relationships that could be construed as a potential conflict of interest.

## Footnotes

1 http://bioinformatics.hsanmartino.it/geena2/

2 http://www.metaboanalyst.ca/

3 https://metlin.scripps.edu/

4 http://motif.gla.ac.uk/Pathos/
