# Supplementary material for: Metabolomic Fingerprints of Individual Algal Cells Using the Single-Probe Mass Spectrometry Technique
Source: Front Plant Sci. 2018 Apr 30;9:571. doi: 10.3389/fpls.2018.00571 (PMC5936784; doi:10.3389/fpls.2018.00571)
Supplement: Supplementary file 1 [file Presentation_1.pdf]

SUPPLEMENTAL DATA AND FIGURES

Figure S1.

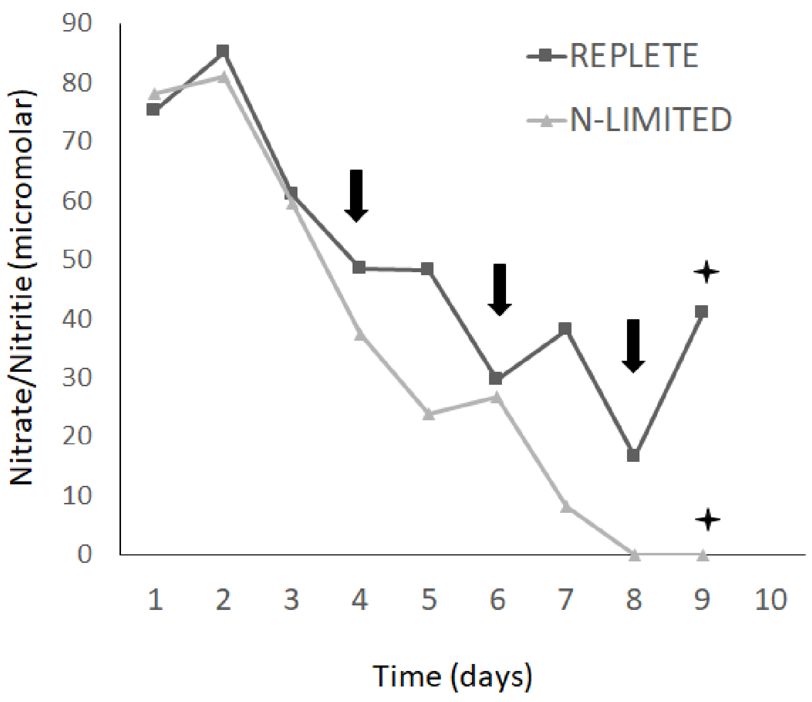

Nitrate+Nitrite concentration in cultures grown for N-limitation experiments. Replete cultures (dark grey line) were fed additional nitrate by adding from sterilized 10 mM $NaNO_3$ stock (black arrows). Nitrate concentrations were reconstituted to ~50 µM every 48 hours to prevent complete depletion of nitrate in the replete culture. The N-limited culture (light grey line) did not receive additional nitrate. Sampling was conducted on day 9 (denoted by crosses), during the middle of the light period, at least 24 hours after nitrate/nitrite concentrations were below the limit of detection in the N-limited culture.

Figure S2.

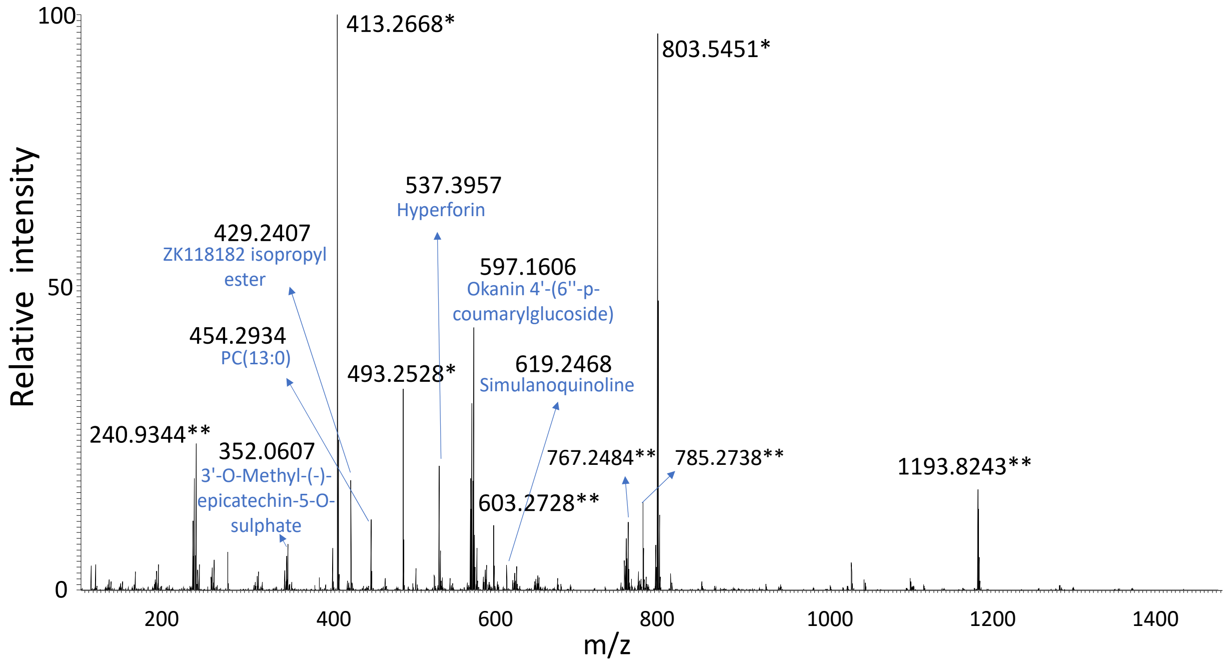

An example mass spectrum of a single *Scrippsiella trochoidea* cell. Peaks are labeled with tentative assignments, background signals (*), or unidentified species (**).

Table S1. List of the 558 metabolites and 23 peptides obtained from MS measurements of single *Scrippsiella trochoidea* cells. Presented are the experimental mass-to-charge ratios (m/z), tentatively assigned names, chemical formulas, mass accuracies (ppm), IDs in METLIN, and format of ions detected during the experiment.

m/z	Name	Chemical formula	Delta mass (ppm)	METLIN ID	Adduct
745.628	Myristin	$C_{45}H_{86}O_6$	4	34484	$[M+Na]^+$
831.489	Hebevinoside I	$C_{44}H_{72}O_{13}$	2	90615	$[M+Na]^+$
717.594	SM(d18:1/17:0)	$C_{40}H_{81}N_2O_6P$	4	83745	$[M+H]^+$
972.724	PI-Cer(d20:0/26:0)	$C_{52}H_{104}NO_{11}P$	1	103158	$[M+Na]^+$
599.248	Magnesium protoporphyrin monomethyl ester	$C_{35}H_{34}MgN_4O_4$	3	63927	$[M+H]^+$
719.524	PG(O-16:0/17:2)	$C_{39}H_{75}O_9P$	2	79820	$[M+H]^+$
919.363	alpha-Amanitin	$C_{39}H_{54}N_{10}O_{14}S$	1	66975	$[M+H]^+$
356.351	Eicosanoyl-EA	$C_{22}H_{45}NO_2$	3	3723	$[M+H]^+$
363.234	9alpha-Fluoro-11beta-hydroxy-6alpha-methylpregn-4-ene-3,20-dione	$C_{22}H_{31}FO_3$	2	70219	$[M+H]^+$
191.982	chlorzoxazone	$C_7H_4ClNO_2$	1	3960	$[M+Na]^+$
593.13	Protoleucomelone	$C_{30}H_{24}O_{13}$	1	90154	$[M+H]^+$
703.545	2-demethylmenaquinone-8	$C_{50}H_{70}O_2$	1	53845	$[M+H]^+$
617.343	Glycosyl-4,4'-diaponeurosporenoate	$C_{36}H_{50}O_7$	3	64107	$[M+Na]^+$
933.449	Betavulgaroside X	$C_{46}H_{70}O_{18}$	3	90220	$[M+Na]^+$
591.258	SWIETENINE	$C_{32}H_{40}O_9$	2	43669	$[M+Na]^+$
207.977	6-hydroxy Chlorzoxazone	$C_7H_4ClNO_3$	1	64664	$[M+Na]^+$
158.117	Tranexamic acid	$C_8H_{15}NO_2$	3	2893	$[M+H]^+$
699.411	Prostaglandin D2-biotin	$C_{36}H_{60}N_4O_6S$	2	44944	$[M+Na]^+$
591.131	5,7,3',5'-Tetrahydroxy-3,6,8,4'-tetramethoxyflavone 3'-glucoside	$C_{25}H_{28}O_{15}$	1	51768	$[M+Na]^+$
653.294	Haplophytine	$C_{37}H_{40}N_4O_7$	4	67517	$[M+H]^+$
611.295	Endomorphin-1	$C_{34}H_{38}N_6O_5$	4	58308	$[M+H]^+$
189.983	2,6-Dichlorobenzamide	$C_7H_5Cl_2NO$	4	68701	$[M+H]^+$
599.136	Vitexin 6''-(3-hydroxy-3-methylglutarate)	$C_{27}H_{28}O_{14}$	1	48665	$[M+Na]^+$
849.233	Pradimicin C	$C_{39}H_{42}N_2O_{18}$	0	66546	$[M+Na]^+$
691.272	Agrimol C	$C_{36}H_{44}O_{12}$	0	68479	$[M+Na]^+$

325.1	Bicozamycin	$C_{12}H_{18}N_2O_7$	1	68945	$[M+Na]^+$
885.632	DISBAC10	$C_{51}H_{88}N_4O_4S_2$	0	64847	$[M+H]^+$
802.534	PC(16:0/20:5)	$C_{44}H_{78}NO_8P$	2	39373	$[M+Na]^+$
874.534	PC(20:5/22:6)	$C_{50}H_{78}NO_8P$	1	39801	$[M+Na]^+$
409.237	Methyl bixin/ (Bixin dimethyl ester)	$C_{26}H_{32}O_4$	0	41462	$[M+H]^+$
897.331	Chlorhexidine gluconate	$C_{34}H_{54}Cl_2N_{10}O_{14}$	4	66788	$[M+H]^+$
423.145	Enantiomultijugin	$C_{24}H_{22}O_7$	2	48589	$[M+H]^+$
655.369	Cuscutic resinoside A	$C_{32}H_{56}O_{12}$	3	102932	$[M+Na]^+$
973.722	TG(20:4/20:4/20:4)	$C_{63}H_{98}O_6$	3	38338	$[M+Na]^+$
909.711	Archaetidylglycerol-myo-inositol	$C_{50}H1_{01}O_{11}P$	4	81171	$[M+H]^+$
262.915	Picloram	$C_6H_3Cl_3N_2O_2$	0	69959	$[M+Na]^+$
678.412	PE(12:0/18:4)	$C_{35}H_{62}NO_8P$	2	76604	$[M+Na]^+$
620.252	5'-Hydroxystreptomycin	$C_{21}H_{39}N_7O_{13}$	3	71782	$[M+Na]^+$
317.087	Ginnalin B	$C_{13}H_{16}O_9$	0	984969	$[M+H]^+$
196.003	Piloty's Acid	$C_6H_7NO_3S$	4	44964	$[M+Na]^+$
493.112	Pongamoside B	$C_{24}H_{22}O_{10}$	3	48493	$[M+Na]^+$
757.631	TG(12:0/12:0/19:1)	$C_{46}H_{86}O_6$	0	98503	$[M+Na]^+$
180.102	Phenacetine	$C_{10}H_{13}NO_2$	0	486	$[M+H]^+$
847.573	PI(O-18:0/18:3)	$C_{45}H_{83}O_{12}P$	4	81014	$[M+H]^+$
539.124	Ketoconazole Metabolite	$C_{25}H_{26}Cl_2N_4O_4$	3	903	$[M+Na]^+$
523.084	5-Carboxypyranopelargonidin 3-O-beta-glucopyranoside	$C_{24}H_{20}O_{12}$	1	47205	$[M+Na]^+$
677.203	Saccatoside	$C_{30}H_{38}O_{16}$	3	985383	$[M+Na]^+$
399.18	Arg Val Cys	$C_{14}H_{28}N_6O_4S_1$	3	18745	$[M+H]^+$
748.547	PS(O-20:0/14:1)	$C_{40}H_{78}NO_9P$	2	78691	$[M+H]^+$
774.51	PC(18:4/18:4)	$C_{44}H_{72}NO_8P$	4	39700	$[M+H]^+$
663.314	N1,N5,N10-Tris-trans-p-coumaroylspermine	$C_{37}H_{44}N_4O_6$	1	94504	$[M+Na]^+$
783.647	TG(13:0/16:1/16:1)	$C_{48}H_{88}O_6$	0	98586	$[M+Na]^+$
605.431	Spheroidenone	$C_{41}H_{58}O_2$	3	41378	$[M+Na]^+$
647.254	Kanokoside D	$C_{27}H_{44}O_{16}$	2	71687	$[M+Na]^+$
375.25	9,11-methane-epoxy PGF1α	$C_{21}H_{36}O_4$	1	45093	$[M+Na]^+$
258.915	1,3,5-Trichloro-(2-methylsulfonyl)benzene	$C_7H_5Cl_3O_2S$	0	92090	$[M+H]^+$
424.281	Myxalamid B	$C_{25}H_{39}NO_3$	2	69330	$[M+Na]^+$

800.595	1-(8-[5]-ladderane-octanyl)-2-(8-[3]-ladderane-octanyl)-sn-glycerophosphocholine	$C_{48}H_{82}NO_6P$	0	46678	$[M+H]^+$
407.182	6beta,7alpha,12beta,13beta)-7-Hydroxy-11,16-dioxo-8,14-apianadien-22,6-olide	$C_{23}H_{28}O_5$	2	87633	$[M+Na]^+$
615.498	DG(17:1/17:1/0:0)	$C_{37}H_{68}O_5$	3	4275	$[M+Na]^+$
485.192	Phenylbutazone glucuronide	$C_{25}H_{28}N_2O_8$	0	1843	$[M+H]^+$
805.437	dihydrodigoxin	$C_{41}H_{66}O_{14}$	3	2072	$[M+Na]^+$
417.122	Granisetron metabolite 4 sulfate	$C_{17}H_{22}N_4O_5S$	4	3151	$[M+Na]^+$
705.523	3-Octaprenyl-4-hydroxybenzoate	$C_{47}H_{70}O_3$	1	63991	$[M+Na]^+$
659.328	Ansatrienin A	$C_{36}H_{48}N_2O_8$	3	69304	$[M+Na]^+$
663.237	5-Methyltetrahydropteroyltri-L-glutamate	$C_{25}H_{36}N_8O_{12}$	3	3684	$[M+Na]^+$
631.371	Goshonoside F4	$C_{32}H_{54}O_{12}$	3	93085	$[M+H]^+$
950.742	PI-Cer(d20:0/26:0)	$C_{52}H_{104}NO_{11}P$	0	103158	$[M+H]^+$
529.192	Deoxyloganic acid tetraacetate	$C_{24}H_{32}O_{13}$	0	69145	$[M+H]^+$
787.678	TG(15:0/15:0/15:0)	$C_{48}H_{92}O_6$	0	98480	$[M+Na]^+$
355.065	5-Amino-6-uracil	$C_9H_{15}N_4O_9P$	0	3497	$[M+H]^+$
353.221	2-Pyrrolidinone, 1-ethyl-4-[2-[(2-hydroxyethyl)amino]ethyl]-3,3-diphenyl- (7CI,8CI,9CI)	$C_{22}H_{28}N_2O_2$	3	2448	$[M+H]^+$
679.309	N3'-Acetylneomycin	$C_{25}H_{48}N_6O_{14}$	4	65820	$[M+Na]^+$
595.379	7,8-Didehydroastaxanthin	$C_{40}H_{50}O_4$	1	41315	$[M+H]^+$
785.651	SM(d16:1/24:1)	$C_{45}H_{89}N_2O_6P$	2	62431	$[M+H]^+$
661.234	Kanokoside C	$C_{27}H_{42}O_{17}$	3	71686	$[M+Na]^+$
897.588	PI(O-18:0/22:6)	$C_{49}H_{85}O_{12}P$	3	81068	$[M+H]^+$
563.207	Galβ1-3GalNAcα-Thr	$C_{21}H_{36}N_2O_{14}$	1	3566	$[M+Na]^+$
106.086	N,N-Dimethylethanolamine N-oxide	$C_4H_{11}NO_2$	2	2810	$[M+H]^+$
755.615	TG(13:0/13:0/17:2)	$C_{46}H_{84}O_6$	1	98558	$[M+Na]^+$
771.649	TG(12:0/12:0/20:1)	$C_{47}H_{88}O_6$	2	98505	$[M+Na]^+$
577.157	Vitexin 6''-(3-hydroxy-3-methylglutarate)	$C_{27}H_{28}O_{14}$	3	48665	$[M+H]^+$
639.494	DG(18:2/18:2/0:0)	$C_{39}H_{68}O_5$	2	4371	$[M+Na]^+$
451.248	Azadiradione	$C_{28}H_{34}O_5$	0	985012	$[M+H]^+$
951.742	TG(18:3/20:3/20:3)	$C_{61}H_{100}O_6$	0	37734	$[M+Na]^+$

506.2	6β-Naloxol-3-glucuronide	$C_{25}H_{31}NO_{10}$	4	1422	$[M+H]^+$
759.646	TG(15:1/15:1/15:1)	$C_{48}H_{86}O_6$	4	98481	$[M+H]^+$
355.151	Gibberellin A20	$C_{19}H_{24}O_5$	1	41213	$[M+Na]^+$
402.373	N-propyl-16,16-dimethyl-5Z,8Z,11Z,14Z-docosatetraenoyl amine	$C_{27}H_{47}NO$	0	36715	$[M+H]^+$
493.216	17-trifluoromethylphenyl trinor PGF2α methyl ester	$C_{25}H_{33}F_3O_5$	2	64694	$[M+Na]^+$
533.272	Calotropin	$C_{29}H_{40}O_9$	4	3590	$[M+H]^+$
821.554	PI(O-16:0/18:2)	$C_{43}H_{81}O_{12}P$	0	81064	$[M+H]^+$
900.55	PC(22:6/22:6)	$C_{52}H_{80}NO_8P$	1	39861	$[M+Na]^+$
353.065	12a-Hydroxydolineone	$C_{19}H_{12}O_7$	1	48044	$[M+H]^+$
857.648	Thyroxine sulfate	$C_{15}H_{11}I_4NO_7S$	3	988	$[M+H]^+$
140.903	Chloroform	$CHCl_3$	4	69721	$[M+Na]^+$
355.191	Tephrowatsin A	$C_{22}H_{26}O_4$	1	47376	$[M+H]^+$
769.63	TG(12:0/12:0/20:2)	$C_{47}H_{86}O_6$	2	98506	$[M+Na]^+$
789.455	MGDG(18:5/18:5)	$C_{45}H_{66}O_{10}$	0	80071	$[M+H]^+$
656.304	Rifamycin W	$C_{35}H_{45}NO_{11}$	3	41011	$[M+H]^+$
535.289	25S)-11alpha,20,26-trihydroxyecdysone	$C_{27}H_{44}O_9$	2	57610	$[M+Na]^+$
576.163	Novclobiocin 105	$C_{28}H_{30}ClNO_{10}$	0	64588	$[M+H]^+$
1030.352	Linoleoyl-CoA	$C_{39}H_{66}N_7O_{17}P_3S$	0	5977	$[M+H]^+$
537.123	Laricitrin 3-(6''-acetylglucoside)	$C_{24}H_{24}O_{14}$	1	50892	$[M+H]^+$
859.55	PG(19:0/22:6)	$C_{47}H_{81}O_{10}P$	4	79304	$[M+Na]^+$
782.566	PC(16:0/20:4)	$C_{44}H_{80}NO_8P$	4	39120	$[M+H]^+$
365.193	2,3-dinor, 6-keto-PGF1α	$C_{18}H_{30}O_6$	1	36156	$[M+Na]^+$
441.202	punaglandin 8	$C_{23}H_{33}ClO_6$	4	36428	$[M+H]^+$
597.083	Mangiferin 6'-gallate	$C_{26}H_{22}O_{15}$	3	93890	$[M+Na]^+$
441.297	26,26,26-trifluoro-25-hydroxy-27-norvitamin D3	$C_{26}H_{39}F_3O_2$	1	41965	$[M+H]^+$
653.361	Metocurine	$C_{40}H_{48}N_2O_6$	3	85496	$[M+H]^+$
785.661	TG(13:0/13:0/19:1)	$C_{48}H_{90}O_6$	2	98566	$[M+Na]^+$
493.17	Okanin 3,4,3'-trimethyl ether 4'-glucoside	$C_{24}H_{28}O_{11}$	0	51978	$[M+H]^+$
583.167	Okanin 4'-alpha-L-arabinofuranosyl-glucoside	$C_{26}H_{30}O_{15}$	2	51969	$[M+H]^+$
429.205	Erycristin	$C_{26}H_{30}O_4$	3	48130	$[M+Na]^+$
731.599	Ubiquinol 8	$C_{49}H_{78}O_4$	2	5974	$[M+H]^+$

619.419	19-Methoxypomolic acid 3-arabinoside	$C_{36}H_{58}O_8$	2	89885	$[M+H]^+$
501.171	Dichotosinin	$C_{24}H_{30}O_{10}$	4	47489	$[M+Na]^+$
587.432	12-Deoxyphorbol 13-palmitate	$C_{36}H_{58}O_6$	2	985064	$[M+H]^+$
863.248	Pradimicin A	$C_{40}H_{44}N_2O_{18}$	0	66544	$[M+Na]^+$
351.081	Ser-Thr-OH	$C_{13}H_{16}N_2O_8$	3	65117	$[M+Na]^+$
1085.48	Mithramycin	$C_{52}H_{76}O_{24}$	0	63779	$[M+H]^+$
697.35	Thalicarpine	$C_{41}H_{48}N_2O_8$	2	67863	$[M+H]^+$
531.256	PI(13:0/0:0)	$C_{22}H_{43}O_{12}P$	0	46743	$[M+H]^+$
401.119	Hydroxyvernolide	$C_{19}H_{22}O_8$	4	67733	$[M+Na]^+$
419.131	Aloesol 7-glucoside	$C_{19}H_{24}O_9$	0	95050	$[M+Na]^+$
856.577	LacCer(d18:1/14:0)	$C_{44}H_{83}NO_{13}$	1	83816	$[M+Na]^+$
785.422	Fumonisin FP2	$C_{39}H_{62}NO_{15}$	3	87950	$[M+H]^+$
137.043	3-amino-1H-1,2,4-triazol-5-yl)methanol	$C_3H_6N_4O$	2	96367	$[M+Na]^+$
426.792	2,4-Dibromo-1-(4-bromophenoxy)benzene	$C_{12}H_7Br_3O$	4	92404	$[M+Na]^+$
383.205	Leu His Asn	$C_{16}H_{26}N_6O_5$	3	16850	$[M+H]^+$
523.149	Nap-TyrMe-OH	$C_{28}H_{24}N_2O_7$	2	65087	$[M+Na]^+$
1013.385	Gossypurpurin	$C_{60}H_{56}N_2O_{13}$	0	95363	$[M+H]^+$
619.244	Simulanoquinoline	$C_{37}H_{34}N_2O_7$	0	94891	$[M+H]^+$
104.107	Choline	$C_5H_{13}NO$	0	56	$[M+H]^+$
669.402	Prostaglandin A1-biotin	$C_{35}H_{58}N_4O_5S$	0	45362	$[M+Na]^+$
627.282	Red chlorophyll catabolite	$C_{35}H_{38}N_4O_7$	1	63971	$[M+H]^+$
619.451	Spirilloxanthin	$C_{42}H_{60}O_2$	3	41379	$[M+Na]^+$
302.305	Sphinganine	$C_{18}H_{39}NO_2$	1	395	$[M+H]^+$
397.237	CG 4305	$C_{25}H_{32}O_4$	0	45349	$[M+H]^+$
760.525	PC(18:4/P-16:0)	$C_{42}H_{76}NO_7P$	0	59704	$[M+Na]^+$
713.509	DG(20:5/22:4/0:0)	$C_{45}H_{70}O_5$	3	4617	$[M+Na]^+$
435.153	Trp Asp Asp	$C_{19}H_{22}N_4O_8$	4	18516	$[M+H]^+$
828.549	PC(16:0/22:6)	$C_{46}H_{80}NO_8P$	2	39390	$[M+Na]^+$
471.235	Simvastatin-6'-carboxylic acid	$C_{25}H_{36}O_7$	0	2452	[M+Na]+
212.164	N-Butylscopolamine metabolite	$C_{12}H_{21}NO_2$	2	2399	$[M+H]^+$
327.243	Hydroxystenozole	$C_{21}H_{30}N_2O$	0	70527	$[M+H]^+$
685.433	3-hexanoyl-NBD Cholesterol	$C_{39}H_{58}N_4O_5$	4	64806	$[M+Na]^+$
393.261	1-Naphthylacetylspermine	$C_{22}H_{34}N_4O$	3	69627	$[M+Na]^+$
633.434	Annonisin	$C_{35}H_{62}O_8$	0	87647	$[M+Na]^+$
428.785	Chlordane	$C_{10}H_6Cl_8$	4	69855	$[M+Na]^+$
947.658	PI(20:0/22:2)	$C_{51}H_{95}O_{13}P$	0	80516	$[M+H]^+$

327.103	Imicyafos	$C_{11}H_{21}N_4O_2PS$	4	72275	$[M+Na]^+$
505.254	26,26,26,27,27,27-hexafluoro-25-hydroxy-23,23,24,24-tetradehydrovitamin D3	$C_{27}H_{34}F_6O_2$	0	42019	$[M+H]^+$
953.521	DGDG(18:5/18:4)	$C_{51}H_{78}O_{15}$	2	46646	$[M+Na]^+$
727.533	1-O-beta-D-Glucopyranosyl-2,3-di-Oglycerol	$C_{41}H_{74}O_{10}$	3	87432	$[M+H]^+$
395.243	(3b,6b,8b,12a)-8,12-Epoxy-7(11)-eremophilene-6-angeloyloxy-8,12-dimethoxy-3-ol	$C_{22}H_{34}O_6$	0	88076	$[M+H]^+$
261.132	Pirimicarb	$C_{11}H_{18}N_4O_2$	0	68822	$[M+Na]^+$
493.348	TG(8:0/8:0/8:0)	$C_{27}H_{50}O_6$	3	62029	$[M+Na]^+$
497.359	1α-hydroxy-2β-(3-hydroxyprop vitamin D3	$C_{30}H_{50}O_4$	2	42397	$[M+Na]^+$
694.478	PE(14:1/P-18:1)	$C_{37}H_{70}NO_7P$	0	60329	$[M+Na]^+$
693.38	Fungichromin	$C_{35}H_{58}O_{12}$	2	69556	$[M+Na]^+$
494.36	PC(P-17:0/0:0)	$C_{25}H_{52}NO_6P$	1	40403	$[M+H]^+$
435.238	Glutathionylspermidine	$C_{17}H_{34}N_6O_5S$	0	63639	$[M+H]^+$
571.235	PKC 412	$C_{35}H_{30}N_4O_4$	1	45414	$[M+H]^+$
425.147	His-Phe-OH	$C_{21}H_{20}N_4O_6$	3	65016	$[M+H]^+$
1123.588	Isohomohalichondrin B	$C_{61}H_{86}O_{19}$	3	71405	$[M+H]^+$
829.725	TG(16:0/16:0/16:0)	$C_{51}H_{98}O_6$	0	3857	$[M+Na]^+$
749.535	PG(16:0/18:1)	$C_{40}H_{77}O_{10}P$	3	40844	$[M+H]^+$
777.56	CHAULMOSULFONE	$C_{48}H_{76}N_2O_4S$	0	44616	$[M+H]^+$
755.555	PA(17:0/21:0)	$C_{41}H_{81}O_8P$	1	81407	$[M+Na]^+$
815.709	TG(16:1/16:1/17:1)	$C_{52}H_{94}O_6$	4	4728	$[M+H]^+$
633.301	BQ 123	$C_{31}H_{42}N_6O_7$	0	69112	$[M+Na]^+$
585.427	2-Hexaprenyl-3-methyl-6-methoxy-1,4-benzoquinol	$C_{38}H_{58}O_3$	1	62813	$[M+Na]^+$
391.23	13,14-dihydro-16,16-difluoro Prostaglandin D2	$C_{20}H_{32}F_2O_5$	2	45569	$[M+H]^+$
459.177	4,6'-Epoxyorotiniflavan-4-ol	$C_{26}H_{28}O_6$	1	47390	$[M+Na]^+$
801.594	all-trans decaprenyl phosphate	$C_{50}H_{83}O_4P$	2	53851	$[M+Na]^+$
686.437	PS(P-16:0/12:0)	$C_{34}H_{66}NO_9P$	0	78741	$[M+Na]^+$
819.542	PI(O-16:0/18:3)	$C_{43}H_{79}O_{12}P$	4	81062	$[M+H]^+$
475.249	Flemiphyllin	$C_{30}H_{34}O_5$	2	47705	$[M+H]^+$
425.219	Didrovaltratum	$C_{22}H_{32}O_8$	4	41153	$[M+H]^+$
325.158	Arg Ala Gly	$C_{11}H_{22}N_6O_4$	4	15649	$[M+Na]^+$

838.556	PS(18:0/22:5)	$C_{46}H_{80}NO_{10}P$	3	61543	$[M+H]^+$
231.099	1-Cyclohexene-1-acrylic acid, 2,6,6-trimethyl-3-oxo-	$C_{12}H_{16}O_3$	0	2291	$[M+Na]^+$
690.508	PC(18:0/11:1)	$C_{37}H_{72}NO_8P$	1	39476	$[M+H]^+$
729.591	N-Oleoyl-D-sphingomyelin	$C_{41}H_{81}N_2O_6P$	0	437	$[M+H]^+$
330.336	N,N-dimethyl-Safingol	$C_{20}H_{43}NO_2$	1	53956	$[M+H]^+$
749.288	Methanofuran	$C_{34}H_{44}N_4O_{15}$	0	63295	$[M+H]^+$
406.293	AX 048	$C_{22}H_{41}NO_4$	0	64840	$[M+Na]^+$
374.302	Anandamide (20:2, n-6)	$C_{22}H_{41}NO_2$	2	36738	$[M+Na]^+$
814.631	PC(16:0/22:2)	$C_{46}H_{88}NO_8P$	1	39381	$[M+H]^+$
889.581	PI(16:0/20:0)	$C_{45}H_{87}O_{13}P$	3	61170	$[M+H]^+$
465.37	4,4-Dimethyl-14a-hydroxymethyl-5a-cholesta-8,24-dien-3b-ol	$C_{30}H_{50}O_2$	0	6102	$[M+Na]^+$
949.71	Hydroxy Linoleins	$C_{57}H_{98}O_9$	0	96530	$[M+Na]^+$
583.281	2'-Norberbamunine	$C_{35}H_{38}N_2O_6$	1	64379	$[M+H]^+$
783.591	PA(18:0/22:0)	$C_{43}H_{85}O_8P$	4	40929	$[M+Na]^+$
587.392	Ilexolide A	$C_{35}H_{54}O_7$	3	67303	$[M+H]^+$
609.328	Boc-Phe(NMe)-Pro-Phe(NMe)-Gly-OMe	$C_{33}H_{44}N_4O_7$	0	65475	$[M+H]^+$
565.386	PA(14:0/12:0)	$C_{29}H_{57}O_8P$	0	82099	$[M+H]^+$
393.298	Chenodeoxycholic Acid	$C_{24}H_{40}O_4$	4	207	$[M+H]^+$
834.473	Rhizonin A	$C_{42}H_{65}N_7O_9$	0	87039	$[M+Na]^+$
200.859	Pentachloroethane	C_2HCl_5	1	73188	[M+H]+
463.188	punaglandin 8	$C_{23}H_{33}ClO_6$	4	36428	$[M+Na]^+$
535.414	(3b,24R,25x)-26-Benzoyloxystigmast-5-ene-3-ol	$C_{36}H_{54}O_3$	1	90018	$[M+H]^+$
869.629	PG(21:0/22:4)	$C_{49}H_{89}O_{10}P$	2	79526	$[M+H]^+$
579.509	38:4(23Z,26Z,29Z,32Z)	$C_{38}H_{68}O_2$	3	74366	$[M+Na]^+$
495.343	26,27-diethyl-1α,25-dihydroxy-20,21-didehydro-23-oxavitamin D3 / 26,27-diethyl-1α,25-dihydroxy-20,21-didehydro-23-oxacholecalciferol	$C_{30}H_{48}O_4$	2	42391	$[M+Na]^+$
481.248	N-(4-benzenesulfonamide) arachidonoyl amine	$C_{26}H_{38}N_2O_3S$	3	36718	$[M+Na]^+$
313.213	5α-dihydrotestosterone	$C_{19}H_{30}O_2$	2	2789	$[M+Na]^+$
573.158	Licuroside	$C_{26}H_{30}O_{13}$	0	51829	$[M+Na]^+$
692.382	Fumonisin C2	$C_{33}H_{57}NO_{14}$	4	53928	$[M+H]^+$

759.605	2-Methyl-6-solanyl-1,4-benzoquinol	$C_{52}H_{80}O_2$	0	64000	$[M+Na]^+$
369.156	Cys Lys Pro	$C_{14}H_{26}N_4O_4S_1$	1	15938	$[M+Na]^+$
571.161	Isoaurasperone A	$C_{32}H_{26}O_{10}$	1	89308	$[M+H]^+$
749.52	20-Deoxynarasin	$C_{43}H_{72}O_{10}$	0	86986	$[M+H]^+$
591.495	DG(16:0/16:0/0:0)	$C_{35}H_{68}O_5$	1	4255	$[M+Na]^+$
535.168	10S,11R-epoxy-punaglandin 3	$C_{25}H_{33}ClO_9$	4	36422	$[M+Na]^+$
473.215	7a-Ethynylestradiol 3-glucuronide	$C_{26}H_{32}O_8$	4	1162	$[M+H]^+$
345.206	11-Dehydrocorticosterone	$C_{21}H_{28}O_4$	0	7003	$[M+H]^+$
587.485	3',4'-Dihydrorhodovibrin	$C_{41}H_{62}O_2$	4	41394	$[M+H]^+$
955.753	Triricinolein	$C_{57}H_{104}O_9$	4	92832	$[M+Na]^+$
885.787	TG(17:0/17:0/18:0)	$C_{55}H_{106}O_6$	1	4759	$[M+Na]^+$
858.368	Cabazitaxel	$C_{45}H_{57}NO_{14}$	1	85581	$[M+Na]^+$
661.464	Purpureacin-1	$C_{37}H_{66}O_8$	1	67027	$[M+Na]^+$
443.309	4,4-difluorovitamin D3	$C_{27}H_{42}F_2O$	1	42073	$[M+Na]^+$
483.206	Acarbose (component 2)	$C_{20}H_{34}O_{13}$	2	764	$[M+H]^+$
633.351	16,16-dimethyl Prostaglandin E2 p-phenyl ester	$C_{37}H_{48}N_2O_7$	3	45607	$[M+H]^+$
461.172	punaglandin 7	$C_{23}H_{31}ClO_6$	4	36427	$[M+Na]^+$
355.286	PGF2α Alcohol methyl ether	$C_{21}H_{38}O_4$	4	4252	$[M+H]^+$
741.585	SM(d18:0/17:0)	$C_{40}H_{83}N_2O_6P$	4	83747	$[M+Na]^+$
587.274	JMV3002	$C_{35}H_{34}N_6O_3$	4	45361	$[M+H]^+$
685.326	gamma-L-Glutamyl-butirosin B	$C_{26}H_{48}N_6O_{15}$	1	72001	$[M+H]^+$
927.729	Hydroxy Linoleins	$C_{57}H_{98}O_9$	0	96530	$[M+H]^+$
513.36	11-OXOURSOLIC ACID ACETATE	$C_{32}H_{48}O_5$	4	44498	$[M+H]^+$
553.386	VITAMIN E SUCCINATE	$C_{33}H_{54}O_5$	0	85011	$[M+Na]^+$
463.257	Norverapamil	$C_{26}H_{36}N_2O_4$	0	3010	$[M+Na]^+$
243.109	Molsidomine	$C_9H_{14}N_4O_4$	0	1332	$[M+H]^+$
801.694	TG(12:0/16:0/18:0)	$C_{49}H_{94}O_6$	0	4706	$[M+Na]^+$
636.593	Cer(d16:2/24:0(2OH))	$C_{40}H_{77}NO_4$	0	102996	$[M+H]^+$
288.17	Zolmitriptan	$C_{16}H_{21}N_3O_2$	2	44026	$[M+H]^+$
763.304	Patellamide C	$C_{37}H_{46}N_8O_6S_2$	1	71066	$[M+H]^+$
573.412	PC(O-18:0/O-3:1)	$C_{29}H_{61}NO_6P$	1	40229	$[M+Na]^+$
833.271	D-erythro-L-galacto-Nonulose	$C_{27}H_{54}O_{27}$	4	86613	$[M+Na]^+$
439.194	Ethyl 7-epi-12-hydroxyjasmonate glucoside	$C_{20}H_{32}O_9$	0	91484	$[M+Na]^+$
647.485	Rollinecin A	$C_{37}H_{68}O_7$	1	86979	$[M+Na]^+$
693.446	PA(18:3/18:3)	$C_{39}H_{65}O_8P$	4	40913	$[M+H]^+$

369.094	2,8-Dihydroxy-3,9,10-trimethoxypterocarpan	$C_{18}H_{18}O_7$	1	48158	$[M+Na]^+$
310.875	Propineb	$C_5H_8N_2S_4Zn$	1	72256	$[M+Na]^+$
717.519	2,3-Bis-O-glycerol 1-phosphate	$C_{43}H_{73}O_6P$	3	66230	$[M+H]^+$
622.541	Cer(t18:0/18:0(2OH))	$C_{36}H_{73}NO_5$	4	103030	$[M+Na]^+$
843.74	TG(16:0/16:0/17:0)	$C_{52}H_{100}O_6$	1	4718	$[M+Na]^+$
417.28	Istamycin A3	$C_{18}H_{36}N_6O_5$	4	71985	$[M+H]^+$
404.065	CELECOXIB	$C_{17}H_{14}F_3N_3O_2S$	0	44362	$[M+Na]^+$
491.206	Tyr Arg Met	$C_{20}H_{32}N_6O_5S_1$	2	15670	$[M+Na]^+$
105.07	Styrene	C_8H_8	1	66571	$[M+H]^+$
545.454	31-hydroxy-32,35-anhydrobacteriohopanetetrol	$C_{35}H_{60}O_4$	4	53887	$[M+H]^+$
780.631	GlcCer(d18:0/20:0)	$C_{44}H_{87}NO_8$	1	41616	$[M+Na]^+$
249.134	CYCLOPENTYLTHEOPHYLLINE	$C_{12}H_{16}N_4O_2$	2	44461	$[M+H]^+$
337.229	Fentanyl	$C_{22}H_{28}N_2O$	4	2695	$[M+H]^+$
645.469	Rolliniastatin-1	$C_{37}H_{66}O_7$	1	46760	$[M+Na]^+$
637.3	Neomycin B	$C_{23}H_{46}N_6O_{13}$	2	1486	$[M+Na]^+$
425.121	p-Hydroxyphenobarbital glucuronide	$C_{18}H_{20}N_2O_{10}$	4	1822	$[M+H]^+$
503.207	LY223982	$C_{30}H_{30}O_7$	1	45208	$[M+H]^+$
501.299	Etioporphyrin III	$C_{32}H_{38}N_4$	0	5589	$[M+Na]^+$
533.453	Propylene glycol mono- and diesters of fats and fatty acids	$C_{32}H_{62}O_4$	1	88494	$[M+Na]^+$
329.244	Eicosatrienoic acid	$C_{20}H_{34}O_2$	3	259	$[M+Na]^+$
405.233	STS-135	$C_{24}H_{31}FN_2O$	4	85218	$[M+Na]^+$
743.524	(2'S)-Deoxymyxol 2'-(2,4-di-O-methyl-α-L-fucoside)	$C_{48}H_{70}O_6$	0	64116	$[M+H]^+$
399.326	(22E)-1α-hydroxy-22,23-didehydrovitamin D3	$C_{27}H_{42}O_2$	0	42087	$[M+H]^+$
935.537	Ritterazine A	$C_{54}H_{76}N_2O_{10}$	2	73530	$[M+Na]^+$
439.32	24,25-hydroxyvitamin D3	$C_{27}H_{44}O_3$	3	220	$[M+Na]^+$
549.414	cholesteryl beta-D-glucoside	$C_{33}H_{56}O_6$	1	57618	$[M+H]^+$
317.148	Ile Tyr	$C_{15}H_{22}N_2O_4$	2	23691	$[M+Na]^+$
349.178	Acitretin	$C_{21}H_{26}O_3$	1	564	$[M+Na]^+$
755.256	Labadoside	$C_{38}H_{42}O_{16}$	1	91539	$[M+H]^+$
781.402	25-O-Desacetylrifampin	$C_{41}H_{56}N_4O_{11}$	0	2315	$[M+H]^+$
335.125	BENZYLBUTYLPHTHALATE	$C_{19}H_{20}O_4$	1	44515	$[M+Na]^+$
795.647	TG(12:0/12:0/22:3)	$C_{49}H_{88}O_6$	0	98514	$[M+Na]^+$
637.391	Evasterioside D	$C_{33}H_{58}O_{10}$	1	84929	$[M+Na]^+$
385.236	Megestrol acetate	$C_{24}H_{32}O_4$	3	1120	$[M+H]^+$

587.296	3-O-acetylecdysone 2-phosphate	$C_{29}H_{47}O_{10}P$	3	57633	$[M+H]^+$
487.231	Testosterone glucuronide	$C_{25}H_{36}O_8$	1	2794	$[M+Na]^+$
216.997	S-Acetyl dihydroasparagusic acid	$C_6H_{10}O_3S_2$	2	90974	$[M+Na]^+$
379.281	Heneicosanedioic acid	$C_{21}H_{40}O_4$	2	35988	$[M+Na]^+$
413.266	Pregnan-20-one, 17-(acetyloxy)-3-hydroxy-6-methyl-, (3b,5b,6a)-	$C_{24}H_{38}O_4$	0	1089	$[M+Na]^+$
429.126	5-2-6-methyl-4-1,4-dihydropyridine-3-carboxylic acid	$C_{19}H_{22}N_2O_8$	1	1557	$[M+Na]^+$
677.351	Cucurbitacin I 2-glucoside	$C_{36}H_{52}O_{12}$	3	90961	$[M+H]^+$
381.297	Hexadecyl Acetyl Glycerol	$C_{21}H_{42}O_4$	1	4249	$[M+Na]^+$
297.075	Pseudobaptigenin methyl ether	$C_{17}H_{12}O_5$	2	47578	$[M+H]^+$
661.536	TG(12:0/12:0/12:0)	$C_{39}H_{74}O_6$	2	62030	$[M+Na]^+$
522.596	Tridodecylamine	$C_{36}H_{75}N$	2	92632	$[M+H]^+$
459.255	Astemizole	$C_{28}H_{31}FN_4O$	1	1122	$[M+H]^+$
150.969	1,3-Dichloro-2-propanol	$C_3H_6Cl_2O$	1	70030	$[M+Na]^+$
445.267	D-NMAPPD	$C_{23}H_{38}N_2O_5$	0	44921	$[M+Na]^+$
577.442	TG(10:0/10:0/10:0)	$C_{33}H_{62}O_6$	3	5532	$[M+Na]^+$
617.51	DG(18:1/16:0/0:0)	$C_{37}H_{70}O_5$	2	4258	$[M+Na]^+$
891.512	Avermectin A2b	$C_{48}H_{74}O_{15}$	2	40983	$[M+H]^+$
881.411	thevetin B	$C_{42}H_{66}O_{18}$	3	57780	$[M+Na]^+$
401.265	9-deoxy-9-methylene-16,16-dimethyl -PGE2	$C_{23}H_{38}O_4$	3	36130	$[M+Na]^+$
443.173	Idebenone Metabolite (1,4-Benzenediol	C19H32O8S	4	765	$[M+Na]^+$
427.216	9-Hydroxyrisperidone	$C_{23}H_{27}FN_4O_3$	4	2349	$[M+H]^+$
267.123	(±)-Goniothalesdiol	$C_{14}H_{18}O_5$	1	44860	$[M+H]^+$
433.258	PGF2α-11-acetate methyl ester	$C_{23}H_{38}O_6$	4	36161	$[M+Na]^+$
935.525	(3b,5b,22a,25R)-Furostane-22-methoxy-3,26-diol 3-[glucosyl-(1->2)-glucoside] 26-glucoside	$C_{46}H_{78}O_{19}$	4	89886	$[M+H]^+$
691.464	Butyl 4'-O-butanoyl-6-O-hexadecanoyl-neohesperidoside	$C_{36}H_{66}O_{12}$	1	102929	$[M+H]^+$
799.678	Thyroxine	$C_{15}H_{11}I_4NO_4$	2	439	$[M+Na]^+$

485.302	Gallopamil	$C_{28}H_{40}N_2O_5$	2	2999	$[M+H]^+$
561.453	DG(14:0/18:4/0:0)	$C_{35}H_{60}O_5$	2	58654	$[M+H]^+$
607.417	PM-Toxin A	$C_{33}H_{60}O_8$	1	67059	$[M+Na]^+$
315.183	Granisetron metabolite 4	$C_{17}H_{22}N_4O_2$	4	3150	$[M+H]^+$
521.229	Beclomethasone dipropionate	$C_{28}H_{37}ClO_7$	2	1247	$[M+H]^+$
551.21	SC-1	$C_{27}H_{25}F_3N_8O_2$	4	64674	$[M+H]^+$
531.183	gibberellin A3 O-beta-D-glucoside	$C_{25}H_{32}O_{11}$	1	53646	$[M+Na]^+$
553.458	30-(-2-(O-2-hydroxy-ethane)-3-hydroxy-propane)-hopane	$C_{35}H_{62}O_3$	2	53884	$[M+Na]^+$
283.262	Oleic Acid	$C_{18}H_{34}O_2$	4	190	$[M+H]^+$
240.232	2R,6R,7S,8S)-7-ethyl-2-propyl-1-azaspiro[5.5]undecan-8-ol	$C_{15}H_{29}NO$	0	263668	$[M+H]^+$
449.172	HoPhe-HoPhe-OH	$C_{25}H_{24}N_2O_6$	2	65354	$[M+H]^+$
663.445	3-hexanoyl-NBD Cholesterol	$C_{39}H_{58}N_4O_5$	4	64806	$[M+H]^+$
797.661	TG(16:1/14:0/16:1)	$C_{49}H_{90}O_6$	2	61725	$[M+Na]^+$
357.147	Desmethylthioridazine	$C_{20}H_{24}N_2S_2$	4	2840	$[M+H]^+$
765.508	(2'S)-Deoxymyxol 2'-(2,4-di-O-methyl-α-L-fucoside)	$C_{48}H_{70}O_6$	2	64116	$[M+Na]^+$
559.469	9-POHSA	$C_{34}H_{64}O_4$	1	263594	$[M+Na]^+$
765.258	Acanthoside D	$C_{34}H_{46}O_{18}$	0	68370	$[M+Na]^+$
827.708	TG(16:0/16:0/16:1)	$C_{51}H_{96}O_6$	2	4716	$[M+Na]^+$
627.155	Delphinidin 3-sophoroside	$C_{27}H_{30}O_{17}$	0	47047	$[M+H]^+$
529.331	Neolinderachalcone	$C_{35}H_{44}O_4$	0	52046	$[M+H]^+$
443.228	Ala Ala Phe Ile	$C_{21}H_{32}N_4O_5$	3	103567	$[M+Na]^+$
133.076	Indoleamine	$C_8H_8N_2$	0	65657	$[M+H]^+$
209.103	8-METHYLCAFFEINE	$C_9H_{12}N_4O_2$	1	84980	$[M+H]^+$
253.122	Cimetidine	$C_{10}H_{16}N_6S$	3	1755	$[M+H]^+$
759.388	Marshdimerin	$C_{48}H_{54}O_8$	1	87506	$[M+H]^+$
557.262	Dipiperamide C	$C_{33}H_{36}N_2O_6$	4	94029	$[M+H]^+$
423.201	Ala Trp Phe	$C_{23}H_{26}N_4O_4$	3	15882	$[M+H]^+$
851.561	PI(13:0/22:1)	$C_{44}H_{83}O_{13}P$	3	80079	$[M+H]^+$
703.488	PG(O-16:0/14:0)	$C_{36}H_{73}O_9P$	0	79814	$[M+Na]^+$
300.289	Sphingosine	$C_{18}H_{37}NO_2$	2	392	[M+H]+
449.359	Hexacosanedioic acid	$C_{26}H_{50}O_4$	2	35992	$[M+Na]^+$
597.318	(5b,7a,12a)-(1,3-dihydro-5-nitro-1,3-dioxo-2H-isoindol-2-yl)methyl ester	$C_{33}H_{44}N_2O_8$	1	6668	$[M+H]^+$
409.164	Cys His Lys	$C_{15}H_{26}N_6O_4S_1$	2	16426	$[M+Na]^+$

647.557	DG(16:0/20:0/0:0)	C39H76O5	2	4256	$[M+Na]^+$
727.431	PA(15:1/22:6)	$C_{40}H_{65}O_8P$	0	81355	$[M+Na]^+$
789.598	PG(O-16:0/22:2)	$C_{44}H_{85}O_9P$	3	79827	$[M+H]^+$
715.406	Avermectin B1b monosaccharide	$C_{40}H_{58}O_{11}$	1	63689	$[M+H]^+$
529.275	(5alpha,6beta,14alpha,20R,22R)-5,6,14,20,27-Pentahydroxy-1-oxowith-24-enolide	$C_{28}H_{42}O_8$	4	89071	$[M+Na]^+$
811.677	TG(13:0/17:1/17:1)	$C_{50}H_{92}O_6$	1	98588	$[M+Na]^+$
559.294	FTY720 phenoxy-biotin	$C_{27}H_{44}N_4O_5S$	2	64810	$[M+Na]^+$
935.715	TG(17:2/20:5/22:6)	$C_{62}H_{94}O_6$	2	38401	$[M+H]^+$
256.3	N-Methyldioctylamine	$C_{17}H_{37}N$	0	103466	$[M+H]^+$
1112.644	Didemnin B	$C_{57}H_{89}N_7O_{15}$	4	71383	$[M+H]^+$
425.29	1b,3a,7a,12a-Tetrahydroxy-5b-cholanoic acid	$C_{24}H_{40}O_6$	0	5296	$[M+H]^+$
185.115	(3R,7R)-1,3,7-Octanetriol	$C_8H_{18}O_3$	1	89425	$[M+Na]^+$
125.096	2,4-octadienal	$C_8H_{12}O$	0	36554	$[M+H]^+$
841.725	TG(16:0/16:1/17:0)	$C_{52}H_{98}O_6$	3	4720	$[M+Na]^+$
491.3	Hemibrevetoxin B	$C_{28}H_{42}O_7$	0	73501	$[M+H]^+$
825.693	TG(16:0/16:1/16:1)	$C_{51}H_{94}O_6$	1	4717	$[M+Na]^+$
363.193	D1-2-Hydroxymethylethisterone	$C_{22}H_{28}O_3$	0	1925	$[M+Na]^+$
801.245	Malvidin 3-rutinoside-5-glucoside	$C_{35}H_{44}O_{21}$	0	47147	$[M+H]^+$
787.434	PI(12:0/17:2)	$C_{38}H_{69}O_{13}P$	3	80042	$[M+Na]^+$
808.583	Dioleoylphosphatidylcholine	$C_{44}H_{84}NO_8P$	0	5572	$[M+Na]^+$
491.356	26,27-diethyl-1α,25-dihydroxy-22-thiavitamin D3	$C_{30}H_{50}O_3S$	1	42395	$[M+H]^+$
483.349	Adlupone	$C_{31}H_{46}O_4$	4	88194	$[M+H]^+$
855.741	TG(16:0/16:0/18:1)	$C_{53}H_{100}O_6$	0	4704	$[M+Na]^+$
759.434	Antanapeptin A	$C_{41}H_{60}N_4O_8$	4	65449	$[M+Na]^+$
809.604	PA(20:0/22:1)	$C_{45}H_{87}O_8P$	1	81689	$[M+Na]^+$
405.167	Desmethylnimodipine	$C_{20}H_{24}N_2O_7$	3	1542	$[M+H]^+$
507.223	Limonoate	$C_{26}H_{34}O_{10}$	1	65618	$[M+H]^+$
813.693	TG(16:1/16:1/17:2)	$C_{52}H_{92}O_6$	4	4734	$[M+H]^+$
621.435	Fasciculic acid A	$C_{36}H_{60}O_8$	1	91574	$[M+H]^+$
505.422	Triacontanedioic acid	$C_{30}H_{58}O_4$	1	35993	$[M+Na]^+$
307.182	Fenazaquin	$C_{20}H_{22}N_2O$	4	72502	$[M+H]^+$
766.427	Cethromycin	$C_{42}H_{59}N_3O_{10}$	0	69257	$[M+H]^+$
525.234	Mascaroside	$C_{26}H_{36}O_{11}$	1	67454	$[M+H]^+$

401.157	Thr Cys Arg	$C_{13}H_{26}N_6O_5S_1$	1	15785	$[M+Na]^+$
895.479	Avermectin A1b	$C_{48}H_{72}O_{14}$	2	40985	$[M+Na]^+$
487.417	17beta,21beta-epoxy-16alpha-ethoxyhopan-3beta-ol	$C_{32}H_{54}O_3$	4	53792	$[M+H]^+$
491.232	Gentamicin A	$C_{18}H_{36}N_4O_{10}$	0	65676	$[M+Na]^+$
337.106	DEOXYSAPPANONE B 7,3'-DIMETHYL ETHER	$C_{18}H_{18}O_5$	4	43628	$[M+Na]^+$
413.197	Bisindolylmaleimide I	$C_{25}H_{24}N_4O_2$	0	45523	$[M+H]^+$
833.63	PG(18:0/22:1)	$C_{46}H_{89}O_{10}P$	4	79145	$[M+H]^+$
507.324	N-tert-Butyloxycarbonyl-deacetyl-leupeptin	$C_{23}H_{44}N_6O_5$	4	69125	$[M+Na]^+$
465.273	Ipecac (Psychotrine)	$C_{28}H_{36}N_2O_4$	3	836	$[M+H]^+$
635.45	Annomuricin A	$C_{35}H_{64}O_8$	1	86618	$[M+Na]^+$
773.423	PI(14:1/14:1)	$C_{37}H_{67}O_{13}P$	2	80913	$[M+Na]^+$
751.517	1,26-Hexacosanediol diferulate	$C_{46}H_{70}O_8$	3	87174	$[M+H]^+$
230.247	Xestoaminol C	$C_{14}H_{31}NO$	3	53933	$[M+H]^+$
455.188	Glu Trp Val	$C_{21}H_{28}N_4O_6$	4	15815	$[M+Na]^+$
282.146	Propranolol	$C_{16}H_{21}NO_2$	1	2170	$[M+Na]^+$
527.459	17-Hydroxypancuronium	$C_{34}H_{58}N_2O_2$	3	1695	$[M+H]^+$
286.31	Spisulosine	$C_{18}H_{39}NO$	1	53932	$[M+H]^+$
638.607	Cer(d14:1/26:0(2OH))	$C_{40}H_{79}NO_4$	1	102976	$[M+H]^+$
299.1	7-Methylxanthosine	$C_{11}H_{14}N_4O_6$	4	64465	$[M+H]^+$
839.708	TG(16:1/16:1/17:0)	$C_{52}H_{96}O_6$	2	4723	$[M+Na]^+$
515.373	25-acetoxy-ergosta-3beta,5alpha,6beta-triol	$C_{30}H_{52}O_5$	4	84010	$[M+Na]^+$
803.458	PPA(18:1/18:1)	$C_{39}H_{74}O_{11}P_2$	2	40951	$[M+Na]^+$
545.49	Tetrahydro-6-(2-hydroxy-16,19-dimethylhexacosyl)-4-methyl-2H-pyran-2-one	$C_{34}H_{66}O_3$	0	87364	$[M+Na]^+$
913.817	TG(12:0/20:0/22:0)	$C_{57}H_{110}O_6$	2	4701	$[M+Na]^+$
851.708	TG(16:1/17:1/17:1)	$C_{53}H_{96}O_6$	2	4736	$[M+Na]^+$
633.504	Glycerol 1,3-didodecanoate 2-decanoate	$C_{37}H_{70}O_6$	3	89512	$[M+Na]^+$
700.416	PS(12:0/16:1)	$C_{34}H_{64}NO_{10}P$	0	77713	$[M+Na]^+$
1005.506	Colubrinoside	$C_{50}H_{78}O_{19}$	3	67285	$[M+Na]^+$
175.025	2,5-Dimethyl-3-(methyldithio)furan	$C_7H_{10}OS_2$	2	94734	$[M+H]^+$
720.52	PS(O-16:0/16:1)	$C_{38}H_{74}NO_9P$	3	78654	$[M+H]^+$
794.427	Tylosin B	$C_{39}H_{65}NO_{14}$	3	65632	$[M+Na]^+$

719.395	Momordicoside E	$C_{37}H_{60}O_{12}$	3	90958	$[M+Na]^+$
479.336	Dolicholide	$C_{28}H_{46}O_6$	1	89706	$[M+H]^+$
539.303	Euphorbia factor Ti2	$C_{32}H_{42}O_7$	4	67420	$[M+H]^+$
698.475	PE(17:0/14:1)	$C_{36}H_{70}NO_8P$	2	3867	$[M+Na]^+$
401.215	Cincassiol B	$C_{20}H_{32}O_8$	4	91863	$[M+H]^+$
477.276	Ipecac (Emetamine)	$C_{29}H_{36}N_2O_4$	2	838	$[M+H]^+$
311.126	4-Methylumbelliferyl heptanoate	$C_{17}H_{20}O_4$	1	34517	$[M+Na]^+$
299.199	19-norandrosterone	$C_{18}H_{28}O_2$	2	1436	$[M+Na]^+$
295.165	Ile Tyr	$C_{15}H_{22}N_2O_4$	0	23691	$[M+H]^+$
819.585	α,α'-Trehalose 6-mycolate	$C_{44}H_{82}O_{13}$	2	35960	$[M+H]^+$
611.337	Hydratopyrrhoxanthinol	$C_{37}H_{48}O_6$	4	91852	$[M+Na]^+$
771.455	(23S,24R,25S)-23,24-dihydroxy-spirost-5-en-3beta-yl O-alpha-L-rhamnopyranosyl-(1-2)-beta-D-glucopyranoside	$C_{40}H_{66}O_{14}$	3	84215	$[M+H]^+$
479.244	lithocholic acid sulfate	$C_{24}H_{40}O_6S$	0	210	$[M+Na]^+$
677.411	(2S,3R)-2,3,4-Trihydroxybutyl 4,6-di-O-acetyl-2-O-hexanoyl-3-O-tetradecanoyl-D-mannopyranoside	$C_{34}H_{60}O_{13}$	0	985096	$[M+H]^+$
229.126	(S)-Menthone 8-thioacetate	$C_{12}H_{20}O_2S$	1	92305	$[M+H]^+$
510.231	Hydroxytetrabenazine glucuronide	$C_{25}H_{35}NO_{10}$	4	2804	$[M+H]^+$
853.725	TG(16:0/17:1/17:1)	$C_{53}H_{98}O_6$	0	4731	$[M+Na]^+$
353.27	9,11-methane-epoxy PGF1α	$C_{21}H_{36}O_4$	3	45093	$[M+H]^+$
545.331	Polysorbate 20	$C_{26}H_{50}O_{10}$	2	92150	$[M+Na]^+$
427.335	4,4'-Diapo-zeta-carotene	$C_{30}H_{44}$	3	41397	$[M+Na]^+$
365.108	His-Ser-OH	$C_{15}H_{16}N_4O_7$	3	65146	$[M+H]^+$
328.321	Stearoylethanolamide	$C_{20}H_{41}NO_2$	0	3720	$[M+H]^+$
795.433	1-hexadecanyl-2-((2'-alpha-glucosyl)-beta-glucosyl)-3-beta-xylosyl-sn-glycerol	$C_{36}H_{68}O_{17}$	2	46631	$[M+Na]^+$
743.465	PA(16:0/22:6)	$C_{41}H_{69}O_8P$	3	40934	$[M+Na]^+$
377.161	MS-275	$C_{21}H_{20}N_4O_3$	0	45517	$[M+H]^+$
671.576	Cholesteryl linoleate	$C_{45}H_{76}O_2$	3	5584	$[M+Na]^+$
267.066	Phenanthrene-4,5-dicarboxylate	$C_{16}H_{10}O_4$	3	72096	$[M+H]^+$
422.197	Quinacrine	$C_{23}H_{30}ClN_3O$	0	24079	$[M+Na]^+$
793.568	PA(19:0/22:2)	$C_{44}H_{83}O_8P$	4	81641	$[M+Na]^+$

1111.641	Valinomycin	$C_{54}H_{90}N_6O_{18}$	2	64667	$[M+H]^+$
207.077	Benzhydrol	$C_{13}H_{12}O$	4	1761	$[M+Na]^+$
309.278	11,14-trans-Eicosadienoic acid	$C_{20}H_{36}O_2$	2	24088	$[M+H]^+$
471.185	Ala Asp Glu His	$C_{18}H_{26}N_6O_9$	3	104346	$[M+H]^+$
467.297	Stearyl citrate	$C_{24}H_{44}O_7$	1	88503	$[M+Na]^+$
600.53	Cer(d15:2/22:0)	$C_{37}H_{71}NO_3$	4	102964	$[M+Na]^+$
481.364	1α,25-dihydroxy-24a,24b,24c-trihomovitamin D3	$C_{30}H_{50}O_3$	2	42392	$[M+Na]^+$
327.145	Tetranor-PGEM	$C_{16}H_{22}O_7$	3	3822	$[M+H]^+$
737.45	Antanapeptin A	$C_{41}H_{60}N_4O_8$	2	65449	$[M+H]^+$
176.11	7-Methylthioheptanaldoxime	$C_8H_{17}NOS$	2	64517	$[M+H]^+$
537.277	Ambenonium	$C_{28}H_{42}Cl_2N_4O_2$	2	85469	$[M+H]^+$
707.487	PG(17:0/14:1)	$C_{37}H_{71}O_{10}P$	1	3879	$[M+H]^+$
515.264	20α-Dihydroprogesterone glucuronide	$C_{27}H_{40}O_8$	4	2127	$[M+Na]^+$
309.132	14-Dihydroxycornestin	$C_{16}H_{20}O_6$	4	67011	$[M+H]^+$
675.269	Secoisotetrandrine	$C_{38}H_{40}N_2O_8$	1	95664	$[M+Na]^+$
423.308	(±)1,2-Didecanoyl-glycerol (10:0)	$C_{23}H_{44}O_5$	0	43449	$[M+Na]^+$
780.554	PC(16:0/18:2)	$C_{42}H_{80}NO_8P$	3	39327	$[M+Na]^+$
236.162	N-(9-Oxodecyl)acetamide	$C_{12}H_{23}NO_2$	0	984916	$[M+Na]^+$
421.2	Pregnanolone sulfate	$C_{21}H_{34}O_5S$	4	3557	$[M+Na]^+$
899.711	TG(17:2/17:2/20:3)	$C_{57}H_{96}O_6$	1	36897	$[M+Na]^+$
487.377	26,27-diethyl-1α,25-dihydroxy-20,21-methano-23-oxavitamin D3	$C_{31}H_{50}O_4$	2	42424	$[M+H]^+$
321.244	(±)15-HETE	$C_{20}H_{32}O_3$	4	3836	$[M+H]^+$
251.093	TEBUTHIURON	$C_9H_{16}N_4OS$	2	44583	$[M+Na]^+$
409.311	3,5,3',5'-Tetra-tert-butyldiphenoquinone	$C_{28}H_{40}O_2$	2	2099	$[M+H]^+$
939.84	TG(18:2/20:1/20:1)	$C_{61}H_{110}O_6$	2	37356	$[M+H]^+$
123.117	Santene	C_9H_{14}	1	92881	$[M+H]^+$
293.208	Ambrettolic acid	$C_{16}H_{30}O_3$	2	35483	$[M+Na]^+$
462.745	2,4,5-Tribromo-1-(4-chlorobenzoyl)imidazole	$C_{10}H_4Br_3ClN_2O$	0	68869	$[M+Na]^+$
664.452	PS(P-16:0/12:0)	$C_{34}H_{66}NO_9P$	4	78741	$[M+H]^+$
463.307	3-dehydroecdysone	$C_{27}H_{42}O_6$	3	57617	$[M+H]^+$
449.268	23S)-1α-hydroxy-25,27-didehydrovitamin D3 26,23-lactone	$C_{27}H_{38}O_4$	3	42527	$[M+Na]^+$

437.198	Met Arg Met	$C_{26}H_{32}N_6O_4S_2$	4	15734	$[M+H]^+$
422.303	NSC 23766	$C_{24}H_{35}N_7$	0	45485	$[M+H]^+$
134.117	Bis (2-hydroxypropyl) amine	$C_6H_{15}NO_2$	4	44741	$[M+H]^+$
793.62	SM(d18:2/21:0)	$C_{44}H_{87}N_2O_6P$	0	83764	$[M+Na]^+$
781.632	TG(15:1/15:1/15:1)	$C_{48}H_{86}O_6$	0	98481	$[M+Na]^+$
487.298	N-(2'-(4-benzenesulfonamide)-ethyl) arachidonoyl amine	$C_{28}H_{42}N_2O_3S$	1	36721	$[M+H]^+$
245.083	6-(Pentylthio)purine	$C_{10}H_{14}N_4S$	0	70204	$[M+Na]^+$
206.151	Tecostanine	$C_{11}H_{21}NO$	2	68068	$[M+Na]^+$
311.293	Phytenic acid	$C_{20}H_{38}O_2$	4	201	$[M+H]^+$
877.725	TG(17:1/17:2/18:1)	$C_{55}H_{98}O_6$	3	36897	$[M+H]^+$
717.368	Amataine	$C_{43}H_{48}N_4O_6$	4	66972	$[M+H]^+$
583.329	15(S)-HETE-biotin	$C_{30}H_{48}N_4O_4S$	0	45652	$[M+Na]^+$
735.514	PG(13:0/20:1)	$C_{39}H_{75}O_{10}P$	4	78908	$[M+H]^+$
795.516	PG(16:0/20:3)	$C_{42}H_{77}O_{10}P$	1	61854	$[M+Na]^+$
415.164	O-Desmethylcarvedilol	$C_{23}H_{24}N_2O_4$	2	1571	$[M+Na]^+$
265.155	2H-Indol-2-one, 1,3-dihydro-4-[2-hydroxy-3-[(1-methylethyl)amino]propoxy	$C_{14}H_{20}N_2O_3$	1	1944	$[M+H]^+$
621.109	Cyanidin 3-(3'',6''-dimalonylglucoside)	$C_{27}H_{24}O_{17}$	0	46905	$[M+H]^+$
809.659	TG(14:1/14:1/19:1)	$C_{50}H_{90}O_6$	4	98686	$[M+Na]^+$
461.342	all-trans-Carophyll yellow	$C_{32}H_{44}O_2$	1	88793	$[M+H]^+$
667.127	Coenzyme F420-1	$C_{24}H_{29}N_4O_{15}P$	1	72892	$[M+Na]^+$
792.517	PS(O-18:0/18:4)	$C_{42}H_{76}NO_9P$	2	78678	$[M+Na]^+$
819.511	PG(16:0/22:5)	$C_{44}H_{77}O_{10}P$	4	61857	$[M+Na]^+$
545.058	8-Hydroxyluteolin 8-glucoside-3'-sulfate	$C_{21}H_{20}O_{15}S$	2	49817	$[M+H]^+$
607.385	Neofusapyrone	$C_{34}H_{54}O_9$	1	984931	$[M+H]^+$
403.112	HoPhe-Asp-OH	$C_{19}H_{18}N_2O_8$	3	65054	$[M+H]^+$
457.328	3a,7a-Dihydroxycoprostanic acid	$C_{27}H_{46}O_4$	1	5348	$[M+Na]^+$
455.312	26,26,26-trifluoro-25-hydroxyvitamin D3	$C_{27}H_{41}F_3O_2$	2	42070	$[M+H]^+$
626.537	N-(3-(15-methyl-hexadecanoyloxy)-13-methyl-tetradecanoyl)-L-serine	$C_{37}H_{71}NO_6$	2	45744	$[M+H]^+$
735.457	Hexanoyloxyisomytiloxanthin	$C_{46}H_{64}O_6$	3	41322	$[M+Na]^+$
645.542	DG(18:0/18:1/0:0)	$C_{39}H_{74}O_5$	1	4349	$[M+Na]^+$

110.984	Formylphosphonate	CH_3O_4P	1	63591	$[M+H]^+$
400.19	Cryptopleurine	$C_{24}H_{27}NO_3$	4	68407	$[M+Na]^+$
591.368	33-Deoxy-33-hydroperoxyfurohyperforin	$C_{35}H_{52}O_6$	4	93094	$[M+Na]^+$
901.725	TG(17:2/17:2/20:2)	$C_{57}H_{98}O_6$	0	36849	$[M+Na]^+$
523.304	11-Deoxocucurbitacin I	$C_{30}H_{44}O_6$	1	67210	$[M+Na]^+$
469.328	1α,25-dihydroxy-26,27-dimethyl-20,21-didehydro-23-oxavitamin D3	$C_{28}H_{46}O_4$	1	42260	$[M+Na]^+$
809.229	Hydroxypropionic porphyrin III	$C_{39}H_{38}N_4O_{14}$	1	5696	$[M+Na]^+$
883.773	TG(17:0/17:1/18:0)	$C_{55}H_{104}O_6$	0	4770	$[M+Na]^+$
689.568	Glycerol 1,2-didodecanoate 3-tetradecanoate	$C_{41}H_{78}O_6$	1	87427	$[M+Na]^+$
517.371	Menthol propylene glycol carbonate	$C_{28}H_{52}O_8$	4	94334	$[M+H]^+$
563.463	DG(14:0/16:0/0:0)	$C_{33}H_{64}O_5$	2	58646	$[M+Na]^+$
333.149	1-Dehydro-9-fluoro-11-oxotestololactone	$C_{19}H_{21}FO_4$	1	70857	$[M+H]^+$
793.525	PI(P-16:0/16:1)	$C_{41}H_{77}O_{12}P$	3	81086	$[M+H]^+$
435.344	MG(0:0/22:1/0:0)	$C_{25}H_{48}O_4$	1	62335	$[M+Na]^+$
171.088	SIN-1 Chloride	$C_6H_{10}N_4O_2$	2	1336	$[M+H]^+$
867.747	TG(17:1/18:2/18:2)	$C_{56}H_{98}O_6$	3	4915	$[M+H]^+$
797.625	16:2-Glc-Campesterol	$C_{50}H_{84}O_7$	4	103414	$[M+H]^+$
963.515	2-(1-(3-((5-(oxy)-4-methoxy-6-methyltetrahydro-2H-pyran-2-yl)oxy)-14-hydroxy-10,13-dimethyl-2,3,4,7,8,9,10,11,12,13,14,15,16,17-tetradecahydro-1H-cyclopenta[a]phenanthren-17-yl)ethoxy)-6tetrahydro-2H-pyran-3,4,5-triol	$C_{47}H_{78}O_{20}$	0	985413	$[M+H]^+$
869.557	PI(O-16:0/22:6)	$C_{47}H_{81}O_{12}P$	3	81073	$[M+H]^+$
380.277	N-palmitoyl threonine	$C_{20}H_{39}NO_4$	0	75489	$[M+Na]^+$
903.74	TG(17:2/17:2/20:1)	$C_{57}H_{100}O_6$	1	5066	$[M+Na]^+$
220.167	N-methylundec-10-enamide	$C_{12}H_{23}NO$	0	65467	$[M+Na]^+$
329.191	4-(3,5-Diphenylcyclohexyl)phenol	$C_{24}H_{24}O$	3	69933	$[M+H]^+$
537.3957	Hyperforin	$C_{35}H_{52}O_4$	3	63085	$[M+H]^+$
905.755	TG(17:2/17:2/20:0)	$C_{57}H_{102}O_6$	2	4963	$[M+Na]^+$

405.333	2-hydroxy-15Z-tetracosenoic acid	$C_{24}H_{46}O_3$	2	35582	$[M+Na]^+$
419.294	25-hydroxy-16,17,23,24-tetradehydrovitamin D3	$C_{27}H_{40}O_2$	4	42040	$[M+Na]^+$
803.62	PG(P-20:0/19:1)	$C_{45}H_{87}O_9P$	4	79978	$[M+H]^+$
909.787	TG(17:0/17:2/20:0)	$C_{57}H_{106}O_6$	1	4899	$[M+Na]^+$
740.456	Erythromycin B	$C_{37}H_{67}NO_{12}$	0	2574	$[M+Na]^+$
197.078	2-Propylglutaric acid	$C_8H_{14}O_4$	2	2998	$[M+Na]^+$
615.11	Protoleucomelone	$C_{30}H_{24}O_{13}$	1	90154	$[M+Na]^+$
439.264	Istamycin A3	$C_{18}H_{36}N_6O_5$	0	71985	$[M+Na]^+$
495.26	7,11-Bisdeacetylvaltrate 7-(3-methylpentanoate) 11-(3-hydroxy-3-methylbutanoate)	$C_{26}H_{38}O_9$	2	89454	$[M+H]^+$
839.647	PA(22:0/22:0)	$C_{47}H_{93}O_8P$	3	82111	$[M+Na]^+$
713.412	PA(14:1/22:6)	$C_{39}H_{63}O_8P$	4	81302	$[M+Na]^+$
419.223	Fluorometholone 17-acetate	$C_{24}H_{31}FO_5$	0	70218	$[M+H]^+$
301.142	Phthalic acid Mono-2-ethylhexyl Ester	$C_{16}H_{22}O_4$	3	44802	$[M+Na]^+$
879.742	TG(17:0/17:2/18:1)	$C_{55}H_{100}O_6$	0	4800	$[M+Na]^+$
457.268	6-bromo-23-methyl-tetracosa-5E,9Z-dienoic acid	$C_{25}H_{45}BrO_2$	0	96822	$[M+H]^+$
421.239	CAY10510	$C_{24}H_{33}FO_5$	1	65496	$[M+H]^+$
597.1606	Okanin 4'-(6''-p-coumarylglucoside)	$C_{30}H_{28}O_{13}$	0	51962	$[M+H]^+$
807.447	Astragaloside III	$C_{41}H_{68}O_{14}$	3	67273	$[M+Na]^+$
396.251	Myxalamid D	$C_{23}H_{35}NO_3$	0	69332	$[M+Na]^+$
505.323	Dipyridamole	$C_{24}H_{40}N_8O_4$	3	2341	$[M+H]^+$
838.403	N-(1-Deoxy-1-fructosyl)valine	$C_{33}H_{63}N_3O_{21}$	0	92652	$[M+H]^+$
721.382	Arvenin I	$C_{38}H_{56}O_{13}$	3	985392	$[M+H]^+$
433.365	3-oxohexacosanoic acid	$C_{26}H_{50}O_3$	0	45864	$[M+Na]^+$
463.375	MG(0:0/24:1/0:0)	$C_{27}H_{52}O_4$	1	62342	$[M+Na]^+$
907.77	TG(17:1/17:2/20:0)	$C_{57}H_{104}O_6$	2	4930	$[M+Na]^+$
465.233	Linalool oxide D 3-[apiosyl-(1->6)-glucoside]	$C_{21}H_{36}O_{11}$	0	87620	$[M+H]^+$
376.318	Anandamide (20:l, n-9)	$C_{22}H_{43}NO_2$	1	3722	$[M+Na]^+$
569.261	Trp Trp Arg	$C_{28}H_{34}N_8O_4$	2	17291	$[M+Na]^+$
1121.653	Ganglioside GM3 (d18:0/12:0)	$C_{53}H_{98}N_2O_{21}$	2	62615	$[M+Na]^+$
881.756	TG(17:0/17:2/18:0)	$C_{55}H_{102}O_6$	0	4782	$[M+Na]^+$
713.361	Hordatine A glucoside	$C_{34}H_{48}N_8O_9$	0	86994	$[M+H]^+$

795.621	PA(O-20:0/22:1)	$C_{45}H_{89}O_7P$	3	82213	$[M+Na]^+$
453.246	Tafluprost	$C_{25}H_{34}F_2O_5$	2	44903	$[M+H]^+$
243.146	Prilocaine	$C_{13}H_{20}N_2O$	3	2073	$[M+Na]^+$
362.302	AM3102	$C_{21}H_{41}NO_2$	2	45552	$[M+Na]^+$
314.341	2-amino-14,16-dimethyloctadecan-3-ol	$C_{20}H_{43}NO$	2	53931	$[M+H]^+$
248.162	(8S,Z)-6-((S)-3-hydroxy-2-methylpropylidene)-8-methyloctahydroindolizin-8-ol	$C_{13}H_{23}NO_2$	0	263684	$[M+Na]^+$
889.686	PG(22:0/22:1)	$C_{50}H_{97}O_{10}P$	3	79547	$[M+H]^+$
131.083	4-Vinylcyclohexene	C_8H_{12}	0	73021	$[M+Na]^+$
823.677	beta-hydroarchaetidylglyerol	$C_{46}H_{95}O_9P$	1	4719	$[M+Na]^+$
309.203	Hexadecanedioic acid	$C_{16}H_{30}O_4$	2	5642	$[M+Na]^+$
421.328	Tetracosanedioic acid	$C_{24}H_{46}O_4$	1	35991	$[M+Na]^+$

Table S2. *Data files for 77 *Scrippsiella trochoidea* cells measured in single cell MS experiments. Presented are the experimental conditions, corresponding file name, the number of cells for which data are recorded in each respective file, and the range of acquisition times for individual cells. (Note: .mzML is a standard MS file format that can be viewed using freeware such as mMass (http://www.mmass.org/) and ProteoWizard (http://proteowizard.sourceforge.net/index.shtml)).

Experiment condition	File name (.mzML)	# of cells analyzed	Acquisition time (min)
**Light condition**	Algea cell light 1	6	0.02-0.68; 1.01-1.69; 2.15-2.65; 3.92-4.27; 6.41-6.64; 6.83-7.09
	Algea cell light 2	2	1.17-1.80; 2.07-2.50
	Algea cell light 3	6	3.44-3.99; 4.52-5.32; 6.1-6.67; 10.33-11.23; 12.77-13.57; 20.77-21.28
	Algea cell light 4	4	2.67-3.28; 5.09-5.58; 10.16-10.69; 14.26-14.90
	Algea cell light 5	6	3.96-4.18; 4.62-4.82; 5.36-5.67; 6.16-6.39; 7.07-7.34; 7.52-7.73
**Dark condition**	Algea cell dark 1	2	1.63-1.99; 2.95-3.13
	Algea cell dark 2	8	6.08-6.53; 7.06-7.52; 7.92-8.31; 9.19-9.66; 10.02-10.47; 10.73-11.00; 13.07-13.47, 13.70-14.01
	Algea cell dark 3	7	3.55-4.81; 5.32-6.06; 11.05-11.97; 14.48-15.13;16.06-16.75; 17.14-17.79; 19.18-19.95
	Algea cell dark 4	5	3.77-4.13; 6.44-6.80; 7.10-7.47; 7.91-8.13; 8.59-8.95
**N-limited condition**	N-limited 1	7	0.52-0.95; 1.25-1.64; 8.56-8.93; 9.75-10.13; 11.87-12.40; 18.15-18.64; 16.69-20.12
	N-limited 2	2	0.65-0.98; 4.71-4.96
	N-limited 3	1	0.56-0.65
	N-limited 4	2	4.84-5.18; 5.45-5.87
	N-limited 5	2	0.67-0.89; 2.22-2.52
	N-limited 6	2	0.05-0.26; 1.92-2.11
	Replete1	2	9.01-9.47; 11.79-12.16
	Replete2	1	0.98-1.24
	Replete3	3	1.50-1.62; 2.01-2.37; 2.55-2.82
	Replete4	3	0.83-1.02; 1.17-1.35; 1.73-1.98

**Replete condition**	Replete5	6	0.02-0.65; 1.89-2.48; 5.18-5.79; 9.73-10.79; 14.21-14.86; 16.44-16.91

Table S3. PLS-DA cross-validation results. Presented are the performance measures (R^2 and Q^2) for different numbers of components (comps).

Model	Measure	1 comps	2 comps	3 comps	4 comps	5 comps
Light vs dark	Accuracy	0.97619	0.97619	0.97619	0.97619	0.97619
	R2	0.86165	0.97386	0.99618	0.99883	0.99974
	Q2	0.72835	0.8285	0.84477	0.84725	0.84978
N-limited vs replete	Accuracy	0.9	0.95	0.9	0.9	0.9
	R2	0.79324	0.96464	0.89568	0.89955	0.89994
	Q2	0.59696	0.64163	0.63966	0.64254	0.64297
